# Supplementary material for: Genome-Wide Characterization and Analysis of bHLH Transcription Factors Related to Anthocyanin Biosynthesis in Cinnamomum camphora (‘Gantong 1’)
Source: Int J Mol Sci. 2023 Feb 9;24(4):3498. doi: 10.3390/ijms24043498 (PMC9959432; doi:10.3390/ijms24043498)
Supplement: Supplementary file 1 [file ijms-24-03498-s001.zip › ijms-2176940-supplementary.pdf]

Supplementary Material

1 Supplementary Figures and Tables

1.1 Supplementary Figures

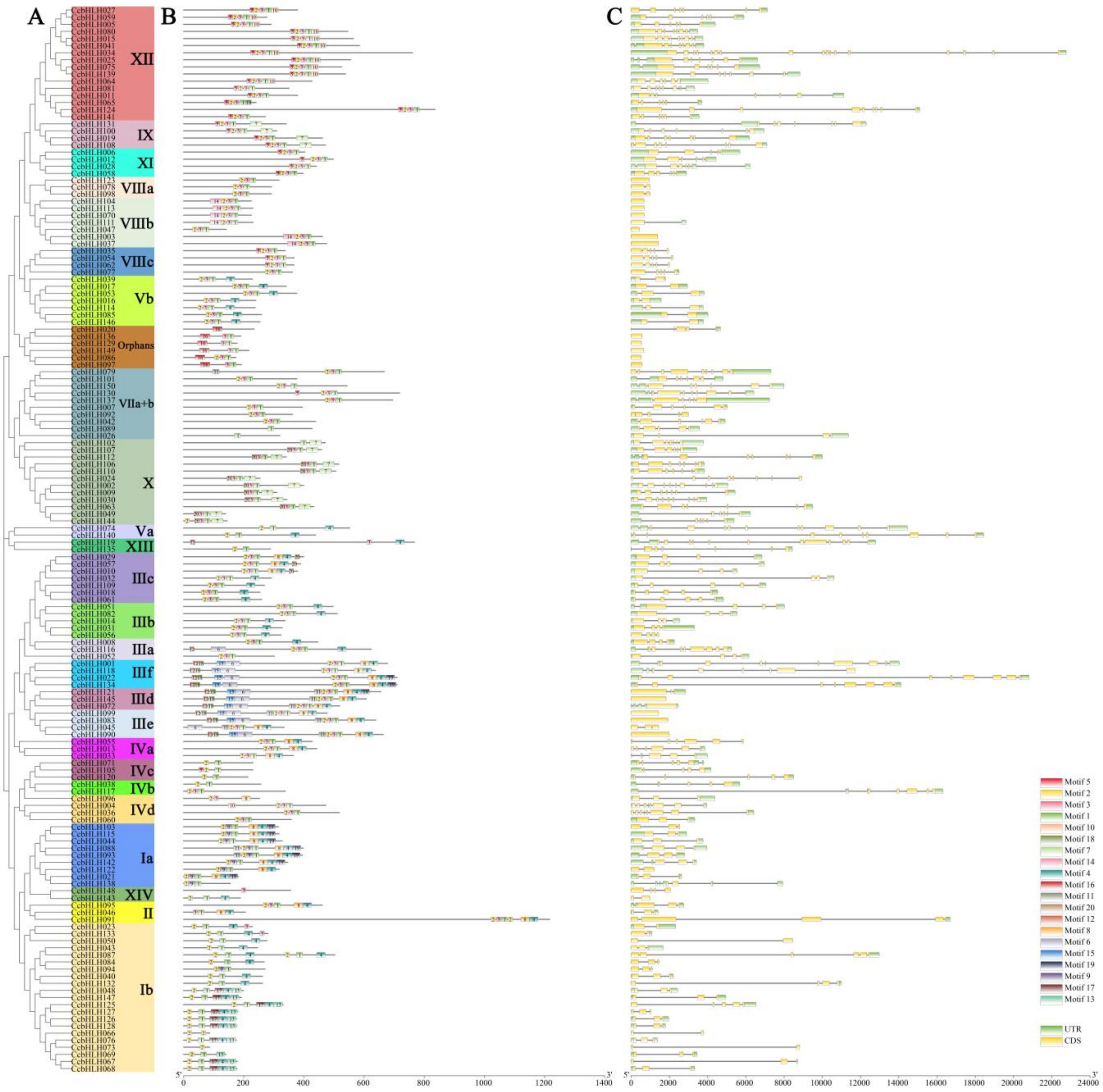

Figure S1. Phylogenetic relationships, conserved motifs, and gene structures of the *CcbHLH* genes. (A) Phylogenetic trees of 150 CcbHLH proteins. The 26 subfamilies are marked with different background colours. (B) Conserved motifs of 150 CcbHLH proteins. Twenty motifs are illustrated by boxes of different colours. (C) Gene structures of 150 *CcbHLH*s. Coding sequence and untranslated regions are shown in yellow and green boxes; introns are shown in black lines.

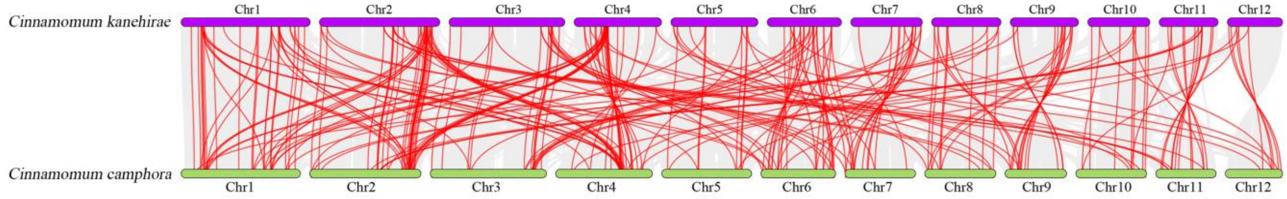

Figure S2. Collinearity analysis of the *bHLH* TF family in *C. camphora* and *C. kanehirae*. Red lines link identified collinear genes.

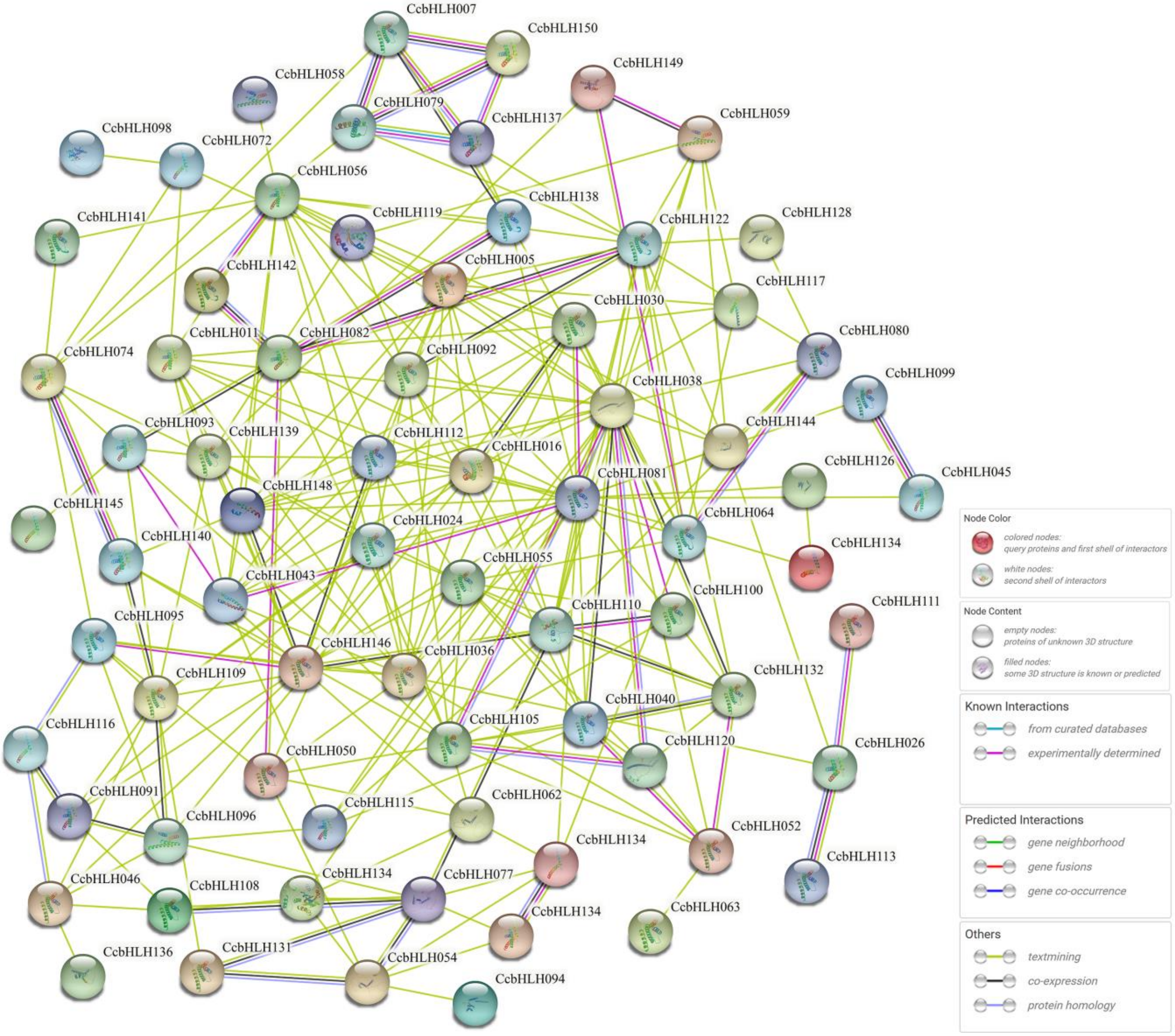

Figure S3. Protein interaction network for CcbHLHs according to CcbHLHs orthologs in *Arabidopsis thaliana*.

1.2 Supplementary Tables

Table S1. Basic protein information of CcbHLH family members

No.	Gene name	Chr	Location		MW (kDa)	pI	I. I.	Protein (aa)	GRAVY
Ccam01G000415	*CcbHLH001*	Chr1	8633570	8647748	76.08	5.26	52.34	677	-0.445
Ccam01G000702	*CcbHLH002*	Chr1	12930089	12935151	43.72	7.2	64.05	398	-0.57
Ccam01G000808	*CcbHLH003*	Chr1	14628373	14629758	50.76	5.76	45.79	461	-0.552
Ccam01G000843	*CcbHLH004*	Chr1	15329593	15333546	54.51	6.48	46.81	472	-0.344
Ccam01G000875	*CcbHLH005*	Chr1	15868845	15873244	31.63	5.62	54.2	290	-0.576
Ccam01G000952	*CcbHLH006*	Chr1	17043099	17048786	44.02	6.11	50.64	402	-0.654
Ccam01G001268	*CcbHLH007*	Chr1	23768793	23773817	43.52	6.67	64.81	394	-0.676
Ccam01G001621	*CcbHLH008*	Chr1	38514404	38516661	49.48	4.99	39.35	446	-0.758
Ccam01G001836	*CcbHLH009*	Chr1	47450836	47456267	33.74	6.66	74.67	309	-0.561
Ccam01G001867	*CcbHLH010*	Chr1	48228951	48234486	42.88	4.79	58.24	378	-0.523
Ccam01G002223	*CcbHLH011*	Chr1	55521306	55532421	41.19	5.44	56.15	378	-0.579
Ccam01G002231	*CcbHLH012*	Chr1	55640969	55645405	55.01	7.14	56.55	497	-0.618
Ccam01G002454	*CcbHLH013*	Chr1	59853203	59857053	49.66	6.68	52.9	442	-0.423
Ccam01G002485	*CcbHLH014*	Chr1	60343673	60346213	38.04	5.07	61.73	336	-0.526
Ccam01G003092	*CcbHLH015*	Chr1	70033610	70037365	60.93	5.64	51.28	564	-0.653
Ccam01G003242	*CcbHLH016*	Chr1	72247038	72248607	26.75	9.21	57.47	239	-0.585
Ccam01G003485	*CcbHLH017*	Chr1	76487044	76489984	37.4	6.2	52.5	340	-0.495
Ccam02G000043	*CcbHLH018*	Chr2	908842	913367	28.9	5.37	53.19	253	-0.528
Ccam02G000340	*CcbHLH019*	Chr2	5985187	5991369	49.3	6.89	62.71	461	-0.634
Ccam02G001398	*CcbHLH020*	Chr2	38979237	38983895	26.48	11.42	75.3	233	-0.766
Ccam02G001693	*CcbHLH021*	Chr2	45180672	45183304	20.15	8.37	67.29	181	-0.086
Ccam02G001706	*CcbHLH022*	Chr2	45365203	45385997	79.78	5.64	59.56	709	-0.611
Ccam02G001730	*CcbHLH023*	Chr2	45830135	45832458	25.39	9.51	56.11	228	-0.56
Ccam02G001915	*CcbHLH024*	Chr2	48708183	48717113	28.39	9.22	53.99	253	-0.714
Ccam02G002109	*CcbHLH025*	Chr2	52039524	52046134	59.33	5.67	49.14	554	-0.549
Ccam02G002686	*CcbHLH026*	Chr2	61901018	61912375	34.81	5.02	54.63	319	-0.619
Ccam02G002773	*CcbHLH027*	Chr2	63379787	63386905	41.12	7.15	48.97	378	-0.499
Ccam02G002908	*CcbHLH028*	Chr2	65370732	65376956	47.64	8.17	47.87	441	-0.552
Ccam02G002983	*CcbHLH029*	Chr2	66483026	66489866	44.37	4.98	62.23	398	-0.493
Ccam02G003003	*CcbHLH030*	Chr2	66819599	66823545	38.06	7.14	65.52	343	-0.494
Ccam02G003014	*CcbHLH031*	Chr2	67027899	67031202	37.03	5.05	58.15	327	-0.455
Ccam02G003024	*CcbHLH032*	Chr2	67224795	67235404	33.01	4.49	70.27	292	-0.621
Ccam02G003029	*CcbHLH033*	Chr2	67296203	67300181	40.29	7.74	57.13	364	-0.409
Ccam02G003063	*CcbHLH034*	Chr2	67829470	67852217	85.28	6.25	51.65	760	-0.535
Ccam02G003130	*CcbHLH035*	Chr2	68734621	68736580	37.58	4.98	42.13	337	-0.605
Ccam02G003212	*CcbHLH036*	Chr2	69942348	69948752	58	7.63	70	517	-0.462
Ccam02G003256	*CcbHLH037*	Chr2	70642905	70644332	51.47	5.56	54.45	475	-0.552
Ccam03G000043	*CcbHLH038*	Chr3	749033	754709	28.16	5.79	55.8	256	-0.68
Ccam03G000529	*CcbHLH039*	Chr3	7684397	7686184	25.51	9.28	49.4	228	-0.434
Ccam03G001506	*CcbHLH040*	Chr3	25046906	25049115	29.24	6.01	72.44	261	-0.484
Ccam03G001581	*CcbHLH041*	Chr3	26751911	26755707	63.53	6.41	55.31	584	-0.719
Ccam03G002509	*CcbHLH042*	Chr3	62978977	62983892	48.27	8.59	58.09	438	-0.617

Ccam03G002787	*CcbHLH043*	Chr3	67684822	67686496	27.33	8.57	52.77	245	-0.417
Ccam03G002879	*CcbHLH044*	Chr3	69167145	69170907	37.08	5.93	59.76	327	-0.435
Ccam03G002889	*CcbHLH045*	Chr3	69301087	69302520	36.53	6.86	52.05	334	-0.322
Ccam03G002937	*CcbHLH046*	Chr3	69983475	69984873	23.44	9.92	37.35	204	-0.445
Ccam03G003221	*CcbHLH047*	Chr3	74526055	74526480	15.85	9.1	86.82	141	-0.634
Ccam04G000139	*CcbHLH048*	Chr4	2160052	2162481	22.73	6.56	46.73	198	-0.51
Ccam04G000570	*CcbHLH049*	Chr4	10374743	10380969	15.61	9.69	53.25	139	-0.361
Ccam04G000814	*CcbHLH050*	Chr4	18031684	18040131	30.82	9.82	47.34	276	-0.587
Ccam04G000873	*CcbHLH051*	Chr4	23141882	23149892	54.32	5.21	60.59	496	-0.505
Ccam04G001130	*CcbHLH052*	Chr4	33259778	33265939	33.73	5.58	45.97	301	-0.304
Ccam04G001284	*CcbHLH053*	Chr4	36996649	37000457	41.02	6.4	53.48	375	-0.543
Ccam04G001648	*CcbHLH054*	Chr4	43092283	43094476	40.39	4.74	54.53	366	-0.645
Ccam04G001694	*CcbHLH055*	Chr4	43680225	43686090	47.98	4.97	47.33	427	-0.566
Ccam04G001699	*CcbHLH056*	Chr4	43765700	43767138	43.15	4.92	58.96	383	-0.607
Ccam04G001717	*CcbHLH057*	Chr4	44075981	44082942	44.11	4.66	67.78	388	-0.508
Ccam04G001759	*CcbHLH058*	Chr4	44628142	44631029	43.4	6.46	48.19	396	-0.558
Ccam04G001815	*CcbHLH059*	Chr4	45561586	45567483	29.6	5.91	56.02	276	-0.657
Ccam04G001837	*CcbHLH060*	Chr4	45974680	45978002	39.88	8.68	65.99	357	-0.459
Ccam04G001949	*CcbHLH061*	Chr4	47535163	47539977	29.06	5.14	57.45	258	-0.576
Ccam04G002165	*CcbHLH062*	Chr4	51029970	51031983	40.3	4.68	58.14	366	-0.645
Ccam04G002172	*CcbHLH063*	Chr4	51168043	51177534	46.97	6.26	50.47	432	-0.404
Ccam04G002219	*CcbHLH064*	Chr4	51851381	51855394	47.65	5.64	52.3	426	-0.615
Ccam04G002418	*CcbHLH065*	Chr4	55009260	55012952	27.24	6.45	57.86	240	-0.572
Ccam04G002640	*CcbHLH066*	Chr4	58124582	58128376	10.12	10.73	57.71	86	-1.016
Ccam04G002642	*CcbHLH067*	Chr4	58148263	58156982	19.93	9.13	54.49	177	-0.238
Ccam04G002643	*CcbHLH068*	Chr4	58173005	58176327	19.68	9.62	50.01	177	-0.229
Ccam04G002644	*CcbHLH069*	Chr4	58193811	58197262	16.05	10.16	45.67	140	-0.574
Ccam05G000235	*CcbHLH070*	Chr5	3652871	3653548	25.61	5.68	68.07	225	-0.692
Ccam05G000742	*CcbHLH071*	Chr5	11705537	11709597	25.64	5.39	59.72	230	-0.724
Ccam05G000818	*CcbHLH072*	Chr5	12992133	12994591	57.14	6.52	41.2	518	-0.367
Ccam05G000902	*CcbHLH073*	Chr5	14968097	14976907	10.04	9.3	45.83	86	-0.595
Ccam05G001253	*CcbHLH074*	Chr5	24204803	24219252	60.56	6.22	52.15	551	-0.822
Ccam05G001269	*CcbHLH075*	Chr5	24722566	24729289	57.68	6.61	51.95	524	-0.53
Ccam05G002154	*CcbHLH076*	Chr5	50150767	50152137	19.81	8.91	57.92	174	-0.265
Ccam05G002171	*CcbHLH077*	Chr5	50400806	50403312	40.37	5.96	47.02	361	-0.594
Ccam05G002316	*CcbHLH078*	Chr5	52967352	52968339	32.4	4.53	56.33	291	-0.352
Ccam05G002354	*CcbHLH079*	Chr5	53433377	53440681	74.18	8.95	61.36	666	-0.592
Ccam06G000067	*CcbHLH080*	Chr6	1026820	1030288	58.66	6.01	47.21	545	-0.59
Ccam06G000318	*CcbHLH081*	Chr6	4585577	4588891	38.82	6.77	60.27	349	-0.67
Ccam06G000343	*CcbHLH082*	Chr6	4990171	4995709	56.04	5.13	58.04	510	-0.545
Ccam06G000478	*CcbHLH083*	Chr6	7089484	7091400	70.41	5.49	49.24	638	-0.578
Ccam06G000723	*CcbHLH084*	Chr6	11121128	11122582	30.44	9.51	55.4	267	-0.348
Ccam06G000831	*CcbHLH085*	Chr6	13424666	13428677	28.22	9.3	57.99	258	-0.526
Ccam06G000990	*CcbHLH086*	Chr6	17311514	17312035	19.76	11.98	70.48	173	-0.639
Ccam06G001416	*CcbHLH087*	Chr6	36923970	36936953	57.1	9.02	63.28	502	-0.736
Ccam06G001588	*CcbHLH088*	Chr6	41635869	41639829	45.07	5.88	67.23	397	-0.651
Ccam06G001856	*CcbHLH089*	Chr6	46323071	46326641	45.71	5.95	54.2	426	-0.528

Ccam06G002000	*CcbHLH090*	Chr6	48263853	48265844	72.27	5.57	51.28	663	-0.459
Ccam06G002056	*CcbHLH091*	Chr6	49116076	49132754	136.68	5.16	49.5	1216	-0.357
Ccam07G000028	*CcbHLH092*	Chr7	431402	434395	39.53	6.31	48.09	361	-0.478
Ccam07G000323	*CcbHLH093*	Chr7	4121727	4124512	44.13	6.36	61.21	394	-0.528
Ccam07G000447	*CcbHLH094*	Chr7	5803846	5804952	30.68	5.83	65.81	270	-0.393
Ccam07G000451	*CcbHLH095*	Chr7	5895892	5898635	51.95	5.07	49.19	460	-0.634
Ccam07G000467	*CcbHLH096*	Chr7	6102541	6106923	28.65	6.48	52.78	252	-0.451
Ccam07G000806	*CcbHLH097*	Chr7	11479838	11480413	21.08	11.56	59.47	191	-0.592
Ccam07G000817	*CcbHLH098*	Chr7	11591427	11592415	32.07	4.52	51.56	291	-0.213
Ccam07G001012	*CcbHLH099*	Chr7	14652722	14654152	52.48	6.49	48.9	476	-0.44
Ccam07G001054	*CcbHLH100*	Chr7	15380875	15387822	33.22	6.2	45.53	309	-0.464
Ccam07G001230	*CcbHLH101*	Chr7	20042699	20048742	41.71	7.7	77.62	376	-0.696
Ccam07G001915	*CcbHLH102*	Chr7	41541957	41545734	51.87	5.2	58.1	470	-0.636
Ccam08G000150	*CcbHLH103*	Chr8	2021192	2023735	35.27	5.52	66.09	315	-0.397
Ccam08G000297	*CcbHLH104*	Chr8	4195816	4196490	24.67	9.44	68.27	224	-0.274
Ccam08G000718	*CcbHLH105*	Chr8	9947275	9951445	25.55	5.89	48.8	230	-0.712
Ccam08G000756	*CcbHLH106*	Chr8	10673989	10677802	57.11	6.08	45.46	515	-0.721
Ccam08G001312	*CcbHLH107*	Chr8	29828625	29832062	50.93	5.83	51.27	458	-0.607
Ccam08G001540	*CcbHLH108*	Chr8	38759329	38766429	50.3	9.46	64.38	471	-0.499
Ccam08G001944	*CcbHLH109*	Chr8	45985682	45992717	30.49	6.1	39.99	268	-0.507
Ccam09G000043	*CcbHLH110*	Chr9	706970	707701	10.04	5.88	74.67	87	-0.594
Ccam09G000250	*CcbHLH111*	Chr9	3585075	3588902	55.65	5.71	42.34	505	-0.722
Ccam09G000335	*CcbHLH112*	Chr9	4652432	4655289	25.29	6.65	66.18	230	-0.498
Ccam09G000592	*CcbHLH113*	Chr9	8580278	8580967	25.19	9.03	58.02	229	-0.357
Ccam09G000671	*CcbHLH114*	Chr9	9896073	9899839	25.59	8.94	62.82	237	-0.416
Ccam09G000703	*CcbHLH115*	Chr9	10536836	10539736	35.31	5.65	66.43	316	-0.465
Ccam09G000849	*CcbHLH116*	Chr9	13089738	13094994	69.72	5.8	41.12	623	-0.729
Ccam09G000924	*CcbHLH117*	Chr9	15046577	15062881	37.96	7.17	64.56	337	-0.73
Ccam09G001546	*CcbHLH118*	Chr9	35440630	35452345	71.92	5.6	59.33	637	-0.478
Ccam09G001725	*CcbHLH119*	Chr9	38936890	38949677	85.06	6.59	43.44	767	-0.237
Ccam09G001727	*CcbHLH120*	Chr9	38978854	38987346	23.93	7.63	58.35	213	-0.657
Ccam10G000132	*CcbHLH121*	Chr10	5335120	5337964	73.04	6.39	45.55	655	-0.475
Ccam10G000299	*CcbHLH122*	Chr10	10557053	10558250	34.94	5.83	71.42	317	-0.524
Ccam10G000671	*CcbHLH123*	Chr10	27257982	27258932	35.07	6.98	69.54	316	-0.349
Ccam10G001192	*CcbHLH124*	Chr10	38491382	38506471	94.85	6.42	46.69	835	-0.413
Ccam10G001273	*CcbHLH125*	Chr10	39898384	39904911	38.25	7.67	56.42	330	-0.352
Ccam10G001274	*CcbHLH126*	Chr10	39913908	39915861	19.82	8.88	52.59	175	-0.549
Ccam10G001275	*CcbHLH127*	Chr10	39934360	39935396	20.05	6.32	52.68	179	-0.234
Ccam10G001276	*CcbHLH128*	Chr10	39958796	39960585	19.86	8.89	46.94	176	-0.407
Ccam10G001702	*CcbHLH129*	Chr10	46143234	46143767	19.98	11.5	69.67	177	-0.51
Ccam10G001809	*CcbHLH130*	Chr10	47900999	47907411	77.19	6.99	61.32	717	-0.569
Ccam11G000202	*CcbHLH131*	Chr11	3196939	3210105	36.36	8.62	57.01	340	-0.298
Ccam11G000280	*CcbHLH132*	Chr11	4276260	4287243	29.23	6.25	57.54	261	-0.333
Ccam11G000711	*CcbHLH133*	Chr11	10293608	10294685	30.84	7.8	63.38	279	-0.661
Ccam11G000722	*CcbHLH134*	Chr11	10485433	10499537	79.31	5.19	61.61	709	-0.553
Ccam11G000731	*CcbHLH135*	Chr11	10601999	10610427	31.01	6.45	43.83	288	-0.246
Ccam11G000873	*CcbHLH136*	Chr11	12886573	12887142	21.07	11.68	69.85	189	-0.539
Ccam11G000993	*CcbHLH137*	Chr11	15174399	15181628	75.55	5.38	62.2	694	-0.537

Ccam11G001086	*CcbHLH138*	Chr11	17412365	17420289	17.15	8.49	69.89	154	-0.356
Ccam11G001256	*CcbHLH139*	Chr11	23687415	23696231	58.36	5.65	49.58	537	-0.516
Ccam11G001278	*CcbHLH140*	Chr11	24739058	24757487	49.24	6.5	52.07	438	-0.774
Ccam11G001488	*CcbHLH141*	Chr11	32381697	32385249	30.65	7.01	47.24	271	-0.672
Ccam11G001570	*CcbHLH142*	Chr11	34521875	34525282	38.92	5.14	62.96	345	-0.429
Ccam11G001635	*CcbHLH143*	Chr11	35920277	35921258	20.69	9.21	40.32	188	-0.188
Ccam12G000639	*CcbHLH144*	Chr12	25049881	25055267	16.09	9.12	46.58	144	-0.394
Ccam12G001023	*CcbHLH145*	Chr12	31336613	31338433	67.49	6.61	50.83	606	-0.626
Ccam12G001109	*CcbHLH146*	Chr12	32507942	32511726	27.45	9.22	48.55	253	-0.287
Ccam12G001241	*CcbHLH147*	Chr12	34739168	34744112	22.07	5.77	42.79	191	-0.512
Ccam12G001317	*CcbHLH148*	Chr12	35723320	35729368	38.85	6.22	38.14	355	-0.385
Ccam12G001327	*CcbHLH149*	Chr12	35840899	35841549	23.75	10.76	61.57	216	-0.854
Ccam12G001429	*CcbHLH150*	Chr12	37399389	37407382	58.97	6.45	68.6	543	-0.641

Table S2. *Ka/Ks* analysis for the *CcbHLH* duplicated genes

Gene 1	Gene 2	*Ka*	*Ks*	*Ka/Ks*
CcbHLH006	*CcbHLH012*	0.23500594	0.56723065	0.41430402
CcbHLH002	*CcbHLH009*	0.09650949	0.53162888	0.18153545
CcbHLH012	*CcbHLH028*	0.23161595	0.90192113	0.25680288
CcbHLH005	*CcbHLH027*	0.22687007	1.21865652	0.18616408
CcbHLH006	*CcbHLH028*	0.2912156	1.03800312	0.28055368
CcbHLH004	*CcbHLH036*	0.43123806	1.09600964	0.39346192
CcbHLH013	*CcbHLH033*	0.15423131	1.00849939	0.15293149
CcbHLH014	*CcbHLH031*	0.20814526	0.99791194	0.20858079
CcbHLH009	*CcbHLH030*	0.16915638	0.99205835	0.17051052
CcbHLH010	*CcbHLH029*	0.38297336	1.77625963	0.21560663
CcbHLH015	*CcbHLH041*	0.14582277	0.66203978	0.22026285
CcbHLH013	*CcbHLH055*	0.2576983	1.09422297	0.23550803
CcbHLH014	*CcbHLH056*	0.26705575	0.92543063	0.28857458
CcbHLH010	*CcbHLH057*	0.33811513	2.40143596	0.14079706
CcbHLH012	*CcbHLH058*	0.28996781	1.00308454	0.28907614
CcbHLH004	*CcbHLH060*	0.49692138	1.49153868	0.33316023
CcbHLH005	*CcbHLH059*	0.20790203	1.09573195	0.18973803
CcbHLH006	*CcbHLH058*	0.37377526	0.98921461	0.37785052
CcbHLH015	*CcbHLH080*	0.2881872	1.1969836	0.2407612
CcbHLH124	*CcbHLH141*	0.19307342	0.56219989	0.34342487
CcbHLH130	*CcbHLH137*	0.34339487	0.86978599	0.39480386
CcbHLH121	*CcbHLH145*	0.22246819	1.54343184	0.14413865
CcbHLH129	*CcbHLH020*	0.55057496	0.97843313	0.56271087
CcbHLH124	*CcbHLH065*	0.3193442	0.93567611	0.3412978
CcbHLH125	*CcbHLH076*	0.35316091	1.02480974	0.34461119
CcbHLH136	*CcbHLH020*	0.31479403	0.60833056	0.51747201
CcbHLH133	*CcbHLH023*	0.13870709	1.19265491	0.11630111
CcbHLH134	*CcbHLH022*	0.13480412	0.73825633	0.18259798
CcbHLH142	*CcbHLH021*	0.39343451	NaN	NaN
CcbHLH147	*CcbHLH048*	0.2723147	0.66388264	0.410185
CcbHLH144	*CcbHLH049*	0.11342323	0.33214852	0.34148346
CcbHLH146	*CcbHLH085*	0.20134274	0.84434473	0.23846035
CcbHLH149	*CcbHLH097*	0.25639202	1.081268	0.23712162
CcbHLH036	*CcbHLH060*	0.28441144	0.78938396	0.36029544
CcbHLH018	*CcbHLH061*	0.0946908	0.92676891	0.10217304
CcbHLH035	*CcbHLH062*	0.2844935	1.0391253	0.27378171
CcbHLH028	*CcbHLH058*	0.19332767	0.5454234	0.35445431
CcbHLH029	*CcbHLH057*	0.24306257	1.06282946	0.22869386
CcbHLH031	*CcbHLH056*	0.14505191	0.60707306	0.2389365
CcbHLH033	*CcbHLH055*	0.17399405	0.52762175	0.32977042
CcbHLH035	*CcbHLH054*	0.21162088	0.5236623	0.40411709
CcbHLH027	*CcbHLH059*	0.10967151	0.65211763	0.16817749
CcbHLH025	*CcbHLH075*	0.25656472	1.28565633	0.19955933
CcbHLH019	*CcbHLH108*	0.22802884	1.25993903	0.18098402
CcbHLH018	*CcbHLH109*	0.1809742	1.48210876	0.12210589

CcbHLH046	*CcbHLH091*	0.27897029	0.59128009	0.47180735
CcbHLH041	*CcbHLH080*	0.31119593	1.35568182	0.22954939
CcbHLH042	*CcbHLH089*	0.22552335	1.01431536	0.22234047
CcbHLH043	*CcbHLH087*	0.31231048	0.60175985	0.51899521
CcbHLH045	*CcbHLH083*	0.24097958	1.86002761	0.12955699
CcbHLH043	*CcbHLH084*	0.38745744	1.3295763	0.29141422
CcbHLH043	*CcbHLH094*	0.40882631	1.29716464	0.31516917
CcbHLH042	*CcbHLH092*	0.37206841	1.07957782	0.34464251
CcbHLH054	*CcbHLH062*	0.3124337	1.12521329	0.27766621
CcbHLH066	*CcbHLH076*	0.43918893	1.02554581	0.42824896
CcbHLH061	*CcbHLH109*	0.19268281	1.21905132	0.15805964
CcbHLH079	*CcbHLH101*	0.2689257	0.87679004	0.30671619
CcbHLH070	*CcbHLH112*	0.23062034	0.99176992	0.23253412
CcbHLH084	*CcbHLH087*	0.40971009	1.55493882	0.26348952
CcbHLH083	*CcbHLH090*	0.19559434	2.25026251	0.08692068
CcbHLH089	*CcbHLH097*	0.32556689	1.18533778	0.2746617
CcbHLH086	*CcbHLH097*	0.22386974	0.91366332	0.24502433
CcbHLH084	*CcbHLH094*	0.20458273	0.83138502	0.24607459
CcbHLH088	*CcbHLH093*	0.23119709	1.19762384	0.1930465
CcbHLH087	*CcbHLH094*	0.44120148	1.24104851	0.35550704
CcbHLH103	*CcbHLH115*	0.19215012	1.16337865	0.16516559
CcbHLH106	*CcbHLH111*	0.2237209	0.93037417	0.24046336
CcbHLH104	*CcbHLH113*	0.17660531	1.28236727	0.1377182

Table S3. *Ka/Ks* analysis for the *CcbHLH* and *Arabidopsis* homologous genes

Cc-Gene	At-Gene	*Ka*	*Ks*	Ka/Ks
CcbHLH001	*AT1G63650.3*	0.391063	NaN	NaN
CcbHLH002	*AT1G51140.1*	0.563993	1.832166	0.307829
CcbHLH003	*AT1G51140.1*	0.540895	3.011273	0.179623
CcbHLH006	*AT1G09530.1*	0.517115	1.834967	0.281812
CcbHLH006	*AT1G09250.1*	0.541174	2.140016	0.252883
CcbHLH009	*AT1G09530.1*	0.549182	NaN	NaN
CcbHLH011	*AT1G51140.1*	0.535489	NaN	NaN
CcbHLH011	*AT1G27740.1*	0.542542	NaN	NaN
CcbHLH012	*AT1G30670.1*	0.589955	NaN	NaN
CcbHLH012	*AT1G68920.4*	0.429324	2.441618	0.175836
CcbHLH013	*AT1G69010.1*	0.372544	3.1349	0.118838
CcbHLH013	*AT1G12860.1*	0.256552	NaN	NaN
CcbHLH014	*AT1G22490.2*	0.42085	NaN	NaN
CcbHLH015	*AT1G22490.2*	0.371546	NaN	NaN
CcbHLH016	*AT2G20100.3*	0.325067	2.473899	0.131399
CcbHLH018	*AT2G42280.3*	0.431623	NaN	NaN
CcbHLH018	*AT2G42300.1*	0.392556	1.99918	0.196358
CcbHLH019	*AT2G20100.3*	0.355311	1.670253	0.212729
CcbHLH020	*AT2G22750.3*	0.451086	2.321151	0.194337
CcbHLH021	*AT2G42280.3*	0.458652	NaN	NaN
CcbHLH028	*AT2G46970.1*	0.650137	NaN	NaN
CcbHLH031	*AT2G22750.3*	0.384004	NaN	NaN
CcbHLH033	*AT2G40200.1*	0.57685	NaN	NaN
CcbHLH033	*AT2G18300.3*	0.53332	2.636851	0.202256
CcbHLH035	*AT2G42280.3*	0.552264	2.426988	0.227551
CcbHLH038	*AT2G22750.3*	0.505538	NaN	NaN
CcbHLH039	*AT2G14760.3*	0.510834	NaN	NaN
CcbHLH041	*AT2G46810.1*	0.434613	NaN	NaN
CcbHLH041	*AT2G46810.1*	0.457829	NaN	NaN
CcbHLH044	*AT2G24260.1*	0.345029	NaN	NaN
CcbHLH045	*AT3G57800.1*	0.412166	3.618364	0.113909
CcbHLH045	*AT3G56770.1*	0.391504	NaN	NaN
CcbHLH047	*AT3G07340.1*	0.429965	NaN	NaN
CcbHLH054	*AT3G21330.1*	0.410367	3.024519	0.13568
CcbHLH055	*AT3G62090.2*	0.711208	1.759927	0.404112
CcbHLH055	*AT3G06590.2*	0.484142	2.0847	0.232236
CcbHLH056	*AT3G05800.1*	0.501939	1.380459	0.363603
CcbHLH058	*AT3G62090.2*	0.668809	3.29868	0.202751
CcbHLH064	*AT3G06590.2*	0.44609	2.631417	0.169525
CcbHLH064	*AT3G06590.2*	0.797705	NaN	NaN
CcbHLH064	*AT3G06120.1*	0.179717	NaN	NaN
CcbHLH074	*AT3G47640.2*	0.475342	2.057972	0.230976
CcbHLH075	*AT3G07340.1*	0.422495	3.631451	0.116343
CcbHLH078	*AT3G07340.1*	0.467619	3.647439	0.128205
CcbHLH080	*AT3G19860.2*	0.587352	NaN	NaN

CcbHLH081	*AT3G23210.1*	0.35224	NaN	NaN
CcbHLH082	*AT4G37850.2*	0.437359	4.531136	0.096523
CcbHLH083	*AT4G30980.2*	0.360209	1.577439	0.22835
CcbHLH083	*AT4G37850.2*	0.403583	NaN	NaN
CcbHLH088	*AT4G29930.3*	0.440754	1.992078	0.221253
CcbHLH089	*AT4G17880.1*	0.314076	NaN	NaN
CcbHLH090	*AT4G37850.2*	0.470054	NaN	NaN
CcbHLH093	*AT4G36540.1*	0.529271	NaN	NaN
CcbHLH093	*AT4G34530.1*	0.404877	NaN	NaN
CcbHLH103	*AT4G17880.1*	0.287119	NaN	NaN
CcbHLH103	*AT4G00050.1*	0.33448	NaN	NaN
CcbHLH105	*AT4G01460.1*	0.425626	NaN	NaN
CcbHLH108	*AT4G29930.3*	0.387341	NaN	NaN
CcbHLH108	*AT4G30980.2*	0.438548	2.920475	0.150163
CcbHLH108	*AT4G36060.3*	0.454389	2.203012	0.206258
CcbHLH109	*AT5G65640.1*	0.377721	NaN	NaN
CcbHLH109	*AT5G53210.1*	0.36693	NaN	NaN
CcbHLH115	*AT5G65640.1*	0.351049	NaN	NaN
CcbHLH115	*AT5G57150.4*	0.292013	NaN	NaN
CcbHLH117	*AT5G46690.1*	0.464377	NaN	NaN
CcbHLH120	*AT5G46760.1*	0.349691	NaN	NaN
CcbHLH122	*AT5G48560.1*	0.462513	NaN	NaN
CcbHLH129	*AT5G01305.1*	0.47317	2.202158	0.214866
CcbHLH129	*AT5G65640.1*	0.395514	NaN	NaN
CcbHLH130	*AT5G50915.2*	0.418551	2.172984	0.192616
CcbHLH130	*AT5G46760.1*	0.381627	NaN	NaN
CcbHLH136	*AT5G46760.1*	0.357134	NaN	NaN
CcbHLH136	*AT5G65320.1*	0.481187	3.750768	0.12829
CcbHLH137	*AT5G57150.4*	0.358213	2.6933	0.133001
CcbHLH137	*AT5G58010.1*	0.341824	NaN	NaN
CcbHLH137	*AT5G65320.1*	0.490928	NaN	NaN

Table S4. *Ka/Ks* analysis for the *CcbHLH* and *Populus trichocarpa* homologous genes

Cc-Gene	Pt-Gene	*Ka*	*Ks*	*Ka/Ks*
CcbHLH001	*PNT53769*	0.291132	1.621532	0.179542
CcbHLH001	*PNT45271*	0.298627	1.57945	0.18907
CcbHLH002	*PNT55309*	0.267573	1.050313	0.254755
CcbHLH002	*PNS93365*	0.189214	0.964579	0.196162
CcbHLH002	*PNT43742*	0.266849	1.013661	0.263253
CcbHLH005	*PNT09940*	0.283679	1.247406	0.227415
CcbHLH005	*PNT00810*	0.301142	1.264235	0.238201
CcbHLH006	*PNT56888*	0.421446	1.414877	0.297868
CcbHLH006	*PNT52177*	0.478165	2.150896	0.22231
CcbHLH006	*PNS97934*	0.426303	1.830828	0.232847
CcbHLH006	*PNT46705*	0.408868	1.529488	0.267323
CcbHLH006	*PNT29960*	0.469848	1.833703	0.256229
CcbHLH006	*PNT19953*	0.395673	1.229559	0.321801
CcbHLH009	*PNT55309*	0.238091	1.103139	0.21583
CcbHLH009	*PNS93365*	0.158024	1.479539	0.106806
CcbHLH009	*PNT43742*	0.247256	1.154567	0.214154
CcbHLH011	*PNS97943*	0.340029	NaN	NaN
CcbHLH011	*PNT29941*	0.331243	NaN	NaN
CcbHLH012	*PNT56888*	0.408461	1.487999	0.274504
CcbHLH012	*PNT52177*	0.604137	2.494412	0.242196
CcbHLH012	*PNS97934*	0.392812	1.962809	0.200128
CcbHLH012	*PNT46705*	0.428952	1.987042	0.215874
CcbHLH012	*PNT29960*	0.364822	1.703151	0.214204
CcbHLH012	*PNT19953*	0.389085	1.228614	0.316686
CcbHLH013	*PNT57202*	0.295426	1.443511	0.204658
CcbHLH013	*PNT20268*	0.30944	1.614281	0.191689
CcbHLH014	*PNT49059*	0.299553	1.430028	0.209473
CcbHLH015	*PNT51556*	0.366357	2.119312	0.172866
CcbHLH017	*PNT16275*	0.201451	4.75474	0.042368
CcbHLH017	*PNT24092*	0.204581	NaN	NaN
CcbHLH018	*PNS94332*	0.355049	2.206933	0.160879
CcbHLH018	*PNT30298*	0.379458	1.695581	0.223792
CcbHLH020	*PNT16564*	0.729359	NaN	NaN
CcbHLH020	*PNT06006*	0.673065	2.103191	0.320021
CcbHLH020	*PNT23859*	0.636333	NaN	NaN
CcbHLH021	*PNT34804*	0.549917	NaN	NaN
CcbHLH021	*PNT25815*	0.0763	1.246864	0.061193
CcbHLH022	*PNT47962*	0.273473	NaN	NaN
CcbHLH022	*PNT37824*	0.261348	NaN	NaN
CcbHLH024	*PNT22856*	0.526702	1.904001	0.276629
CcbHLH025	*PNT16364*	0.281808	1.313722	0.214511
CcbHLH025	*PNT24052*	0.310445	1.369726	0.226648
CcbHLH026	*PNT02672*	0.504749	NaN	NaN
CcbHLH026	*PNT49342*	0.513808	1.993562	0.257733
CcbHLH026	*PNT36648*	0.664318	2.41816	0.27472

CcbHLH027	*PNT00810*	0.285788	1.407075	0.203108
CcbHLH028	*PNT56888*	0.337779	2.558617	0.132016
CcbHLH028	*PNT52177*	0.530757	1.559457	0.340347
CcbHLH028	*PNS97934*	0.375131	1.78794	0.209812
CcbHLH028	*PNT46705*	0.369289	1.795749	0.205646
CcbHLH028	*PNT29960*	0.340489	1.700201	0.200264
CcbHLH028	*PNT19953*	0.309033	2.183737	0.141515
CcbHLH031	*PNT49059*	0.233542	1.561492	0.149563
CcbHLH031	*PNT26394*	0.756511	5.746851	0.131639
CcbHLH033	*PNT57202*	0.273492	1.254739	0.217968
CcbHLH033	*PNT26361*	0.342218	1.71538	0.1995
CcbHLH033	*PNT20268*	0.277505	1.873849	0.148093
CcbHLH034	*PNT57214*	0.196616	1.561241	0.125935
CcbHLH034	*PNT49193*	0.39682	1.550069	0.256001
CcbHLH035	*PNT02498*	0.465504	3.001594	0.155086
CcbHLH035	*PNT49259*	0.392786	2.130418	0.184371
CcbHLH035	*PNT20403*	0.384496	2.031636	0.189255
CcbHLH036	*PNT31782*	0.581434	2.336778	0.248819
CcbHLH039	*PNT17336*	0.386039	1.81905	0.21222
CcbHLH039	*PNT23239*	0.372316	1.631823	0.228159
CcbHLH041	*PNT51556*	0.32612	NaN	NaN
CcbHLH042	*PNT49677*	0.316156	1.437056	0.220002
CcbHLH043	*PNT54427*	0.288898	1.581717	0.182648
CcbHLH043	*PNT44582*	0.305508	1.515289	0.201617
CcbHLH045	*PNT54440*	0.297376	NaN	NaN
CcbHLH045	*PNT44564*	0.280744	NaN	NaN
CcbHLH047	*PNS99213*	0.333148	2.305331	0.144512
CcbHLH047	*PNT30843*	0.370876	NaN	NaN
CcbHLH054	*PNT02498*	0.481968	1.383972	0.34825
CcbHLH054	*PNT49259*	0.40181	1.356559	0.296198
CcbHLH054	*PNT20403*	0.371006	1.733931	0.213968
CcbHLH055	*PNT57202*	0.372709	1.405243	0.265227
CcbHLH055	*PNT26361*	0.416299	1.928535	0.215863
CcbHLH055	*PNT20268*	0.346796	1.56578	0.221485
CcbHLH058	*PNT52177*	0.573407	1.797265	0.319044
CcbHLH058	*PNT56888*	0.426624	2.287698	0.186486
CcbHLH058	*PNS97934*	0.409233	1.575818	0.259696
CcbHLH058	*PNT46705*	0.438571	1.547275	0.283448
CcbHLH058	*PNT29960*	0.407239	1.674664	0.243176
CcbHLH058	*PNT19953*	0.394876	2.36183	0.167191
CcbHLH061	*PNS94332*	0.364544	4.291871	0.084938
CcbHLH061	*PNT30298*	0.391948	2.293331	0.170908
CcbHLH062	*PNT02498*	0.463298	1.462624	0.316758
CcbHLH062	*PNT49259*	0.36966	2.27129	0.162753
CcbHLH062	*PNT20403*	0.340054	1.470811	0.231202
CcbHLH063	*PNT47564*	0.343917	1.663355	0.206761
CcbHLH063	*PNT36309*	0.336662	1.527661	0.220377

CcbHLH063	*PNT38258*	0.319741	1.878868	0.170178
CcbHLH063	*PNT26639*	0.458812	1.938151	0.236727
CcbHLH064	*PNT41429*	0.346664	1.456129	0.238072
CcbHLH064	*PNT36348*	0.32111	1.546873	0.207587
CcbHLH064	*PNT26709*	0.454677	1.583315	0.287168
CcbHLH064	*PNT20883*	0.31226	1.680372	0.185828
CcbHLH065	*PNT09623*	0.346873	1.64448	0.210932
CcbHLH065	*PNT00400*	0.359286	2.251147	0.159601
CcbHLH065	*PNS96429*	0.505078	1.818061	0.277812
CcbHLH065	*PNT40462*	0.490764	2.091415	0.234656
CcbHLH074	*PNT16382*	0.367149	NaN	NaN
CcbHLH074	*PNT40695*	0.402756	1.558423	0.258438
CcbHLH075	*PNT16364*	0.346725	1.937165	0.178986
CcbHLH076	*PNT10047*	0.285073	2.639913	0.107986
CcbHLH076	*PNT00928*	0.326322	NaN	NaN
CcbHLH078	*PNT60249*	0.431184	1.540725	0.279858
CcbHLH078	*PNT13723*	0.430087	1.261472	0.340941
CcbHLH080	*PNT04964*	0.411464	1.828059	0.225082
CcbHLH080	*PNT51292*	0.392598	1.879378	0.208898
CcbHLH080	*PNT51556*	0.416479	2.530465	0.164586
CcbHLH081	*PNT53008*	0.569579	1.830694	0.311128
CcbHLH083	*PNT44564*	0.280272	NaN	NaN
CcbHLH085	*PNT12114*	0.292079	1.270827	0.229834
CcbHLH085	*PNT07935*	0.425225	2.004636	0.212121
CcbHLH085	*PNS91223*	0.416102	1.9122	0.217604
CcbHLH085	*PNT39723*	0.297454	1.405221	0.211678
CcbHLH088	*PNT04113*	0.356634	3.436573	0.103776
CcbHLH088	*PNT50311*	0.373621	2.223603	0.168025
CcbHLH089	*PNT49677*	0.284822	3.771937	0.075511
CcbHLH090	*PNT54440*	0.254681	NaN	NaN
CcbHLH090	*PNT44564*	0.239404	NaN	NaN
CcbHLH092	*PNT49677*	0.512568	2.131443	0.240479
CcbHLH096	*PNT15303*	0.462251	2.509113	0.184229
CcbHLH096	*PNT24984*	0.464114	2.146349	0.216234
CcbHLH097	*PNT37670*	0.765459	3.029412	0.252676
CcbHLH099	*PNT53400*	0.379131	NaN	NaN
CcbHLH099	*PNT04055*	0.3542	NaN	NaN
CcbHLH099	*PNT47736*	0.656523	NaN	NaN
CcbHLH099	*PNT50251*	0.323136	NaN	NaN
CcbHLH099	*PNT45651*	0.394096	NaN	NaN
CcbHLH099	*PNT38069*	0.740982	NaN	NaN
CcbHLH100	*PNT06622*	0.258731	1.341272	0.1929
CcbHLH100	*PNT47794*	0.157252	2.624879	0.059908
CcbHLH100	*PNT38012*	0.152738	2.765409	0.055232
CcbHLH100	*PNT35057*	0.248621	1.653657	0.150346
CcbHLH102	*PNT59457*	0.358156	1.842865	0.194347
CcbHLH102	*PNT11611*	0.361728	1.853335	0.195177
CcbHLH102	*PNT39271*	0.335374	1.696584	0.197676

CcbHLH103	*PNT48909*	0.286953	2.456462	0.116816
CcbHLH103	*PNT35341*	0.297648	NaN	NaN
CcbHLH103	*PNT28057*	0.28943	NaN	NaN
CcbHLH104	*PNT02692*	0.241599	3.204816	0.075386
CcbHLH104	*PNT49349*	0.256411	3.303208	0.077625
CcbHLH104	*PNT36639*	0.273252	2.154249	0.126843
CcbHLH104	*PNT27082*	0.260134	2.100099	0.123867
CcbHLH106	*PNT15681*	0.303597	1.60971	0.188604
CcbHLH106	*PNT24596*	0.34729	1.938602	0.179145
CcbHLH107	*PNT13196*	0.349335	1.448914	0.241101
CcbHLH109	*PNS94332*	0.41992	1.890988	0.222064
CcbHLH109	*PNT30298*	0.409524	2.910811	0.140691
CcbHLH111	*PNT15681*	0.292687	1.49114	0.196284
CcbHLH111	*PNT24596*	0.334365	1.945434	0.171871
CcbHLH112	*PNT35165*	0.306788	NaN	NaN
CcbHLH112	*PNT28262*	0.356424	NaN	NaN
CcbHLH113	*PNT02692*	0.235671	4.031459	0.058458
CcbHLH113	*PNT49349*	0.297668	2.632732	0.113064
CcbHLH113	*PNT36639*	0.317295	1.609263	0.197168
CcbHLH113	*PNT27082*	0.307349	1.774405	0.173212
CcbHLH114	*PNT48979*	0.388233	2.371136	0.163733
CcbHLH115	*PNT48909*	0.283384	2.215878	0.127888
CcbHLH115	*PNT35341*	0.344137	2.40309	0.143206
CcbHLH115	*PNT28057*	0.339934	2.542961	0.133676
CcbHLH117	*PNT41649*	0.331784	1.774579	0.186965
CcbHLH117	*PNT36201*	0.294478	1.608966	0.183023
CcbHLH117	*PNT21113*	0.308071	1.877675	0.16407
CcbHLH118	*PNT03691*	0.405633	1.786786	0.227018
CcbHLH118	*PNT45271*	0.40374	1.659502	0.24329
CcbHLH119	*PNT47713*	0.443658	1.830656	0.242349
CcbHLH119	*PNT38088*	0.450932	1.338625	0.336862
CcbHLH120	*PNT15223*	0.313551	1.766865	0.177462
CcbHLH120	*PNT25060*	0.232143	1.597051	0.145357
CcbHLH121	*PNT03984*	0.334791	2.059049	0.162595
CcbHLH121	*PNT50177*	0.326443	NaN	NaN
CcbHLH122	*PNT09201*	0.233521	2.72777	0.085609
CcbHLH122	*PNT00002*	0.247502	NaN	NaN
CcbHLH124	*PNT09623*	0.350889	1.355321	0.258898
CcbHLH124	*PNT00400*	0.340647	1.621677	0.210059
CcbHLH124	*PNS96429*	0.381108	1.317788	0.289203
CcbHLH124	*PNT40462*	0.374151	1.465643	0.255281
CcbHLH125	*PNT10047*	0.474139	2.309576	0.205293
CcbHLH125	*PNT00928*	0.424055	NaN	NaN
CcbHLH129	*PNT16564*	0.508245	4.104833	0.123816
CcbHLH129	*PNT06006*	0.419297	1.730876	0.242245
CcbHLH129	*PNT34390*	0.434215	2.206879	0.196755
CcbHLH129	*PNT23859*	0.454284	NaN	NaN

CcbHLH130	*PNT04213*	0.624485	1.93177	0.323271
CcbHLH131	*PNT31521*	0.37743	1.870803	0.201748
CcbHLH136	*PNT16564*	0.414036	3.929545	0.105365
CcbHLH136	*PNT50574*	0.916448	NaN	NaN
CcbHLH136	*PNT34390*	0.375408	1.751747	0.214305
CcbHLH136	*PNT23859*	0.395698	2.8209	0.140274
CcbHLH137	*PNT05883*	0.490734	1.40722	0.348726
CcbHLH137	*PNT04213*	0.594475	1.589967	0.373891
CcbHLH137	*PNT34242*	0.465996	1.446159	0.32223
CcbHLH144	*PNT08075*	0.331652	1.406553	0.235791
CcbHLH145	*PNT03984*	0.240688	1.855189	0.129738
CcbHLH145	*PNT50177*	0.252087	3.717722	0.067807
CcbHLH146	*PNT12114*	0.281933	1.235431	0.228206
CcbHLH146	*PNS91223*	0.390369	1.402608	0.278317
CcbHLH146	*PNT39723*	0.336278	1.131326	0.297242
CcbHLH150	*PNT51632*	0.347751	2.106323	0.165099

Table S5. *Ka/Ks analysis for the CcbHLH and C. kanehirae* homologous genes

Cc-Gene	At-Gene	*Ka*	*Ks*	Ka/Ks
CcbHLH001	*CKAN_00040200*	0.005705	0.038992	0.14631
CcbHLH002	*CKAN_00069300*	0.005072	0.02249	0.225512
CcbHLH003	*CKAN_00079900*	0.018835	0.036623	0.514289
CcbHLH004	*CKAN_00083300*	0.01367	0.009783	1.397346
CcbHLH005	*CKAN_00086200*	0.06516	0.105861	0.61553
CcbHLH006	*CKAN_00093200*	0.004264	0.019085	0.223443
CcbHLH007	*CKAN_00123100*	0.011962	0.015847	0.754836
CcbHLH009	*CKAN_00172600*	0.001403	0.028666	0.048927
CcbHLH010	*CKAN_00174500*	0.069818	0.06544	1.066892
CcbHLH011	*CKAN_00209700*	0.009267	0.038668	0.239641
CcbHLH012	*CKAN_00210400*	0.007278	0.009788	0.743589
CcbHLH013	*CKAN_00235600*	0.003876	0.017367	0.223164
CcbHLH014	*CKAN_00238300*	0.001273	0.013647	0.093286
CcbHLH015	*CKAN_00297600*	0.004631	0.025937	0.178528
CcbHLH016	*CKAN_00313300*	0.01326	0.044685	0.296743
CcbHLH017	*CKAN_00336300*	0.001297	0.020409	0.06355
CcbHLH012	*CKAN_00093200*	0.236947	0.513005	0.46188
CcbHLH006	*CKAN_00210400*	0.237585	0.526228	0.451487
CcbHLH002	*CKAN_00172600*	0.094914	0.493676	0.19226
CcbHLH008	*CKAN_00152700*	0.013472	0.035373	0.380839
CcbHLH012	*CKAN_00628900*	0.232751	0.880167	0.26444
CcbHLH005	*CKAN_00615000*	0.211236	1.2797	0.165067
CcbHLH006	*CKAN_00628900*	0.289126	0.989126	0.292305
CcbHLH013	*CKAN_00640400*	0.154198	1.025908	0.150304
CcbHLH014	*CKAN_00639100*	0.206316	0.983611	0.209753
CcbHLH004	*CKAN_00657400*	0.418242	1.208188	0.346173
CcbHLH010	*CKAN_00635900*	0.370651	1.519535	0.243924
CcbHLH015	*CKAN_00851700*	0.153363	0.652913	0.23489
CcbHLH012	*CKAN_01157500*	0.27568	1.005723	0.274111
CcbHLH005	*CKAN_01152000*	0.21399	1.186284	0.180387
CcbHLH006	*CKAN_01157500*	0.371884	1.010547	0.368002
CcbHLH013	*CKAN_01163500*	0.20416	0.997132	0.204748
CcbHLH014	*CKAN_01163000*	0.270309	0.908778	0.297442
CcbHLH010	*CKAN_01161300*	0.342339	2.4434	0.140108
CcbHLH015	*CKAN_01610300*	0.289853	1.224251	0.23676
CcbHLH001	*CKAN_02091900*	0.243859	0.912859	0.267137
CcbHLH129	*CKAN_00486600*	0.355557	0.926623	0.383713
CcbHLH124	*CKAN_01094000*	0.313592	0.945418	0.331696
CcbHLH126	*CKAN_01446500*	0.297966	0.869566	0.342661
CcbHLH124	*CKAN_02314600*	0.038262	0.079585	0.480769

CcbHLH125	*CKAN_02323400*	0.252058	0.459542	0.548498
CcbHLH129	*CKAN_02361800*	0.002533	0.045641	0.055492
CcbHLH130	*CKAN_02372300*	0.004829	0.024959	0.193458
CcbHLH123	*CKAN_02276000*	0.009947	0.060837	0.163501
CcbHLH121	*CKAN_02255000*	0.01791	0.041115	0.435617
CcbHLH122	*CKAN_02242100*	0.008303	0.027234	0.304859
CcbHLH124	*CKAN_02413200*	0.191015	0.538584	0.354662
CcbHLH121	*CKAN_02582300*	0.221462	1.565148	0.141496
CcbHLH136	*CKAN_00486600*	0.201376	0.537351	0.374758
CcbHLH133	*CKAN_00514000*	0.130461	1.335488	0.097688
CcbHLH134	*CKAN_00511800*	0.135023	0.75243	0.17945
CcbHLH142	*CKAN_00510700*	0.380008	NaN	NaN
CcbHLH141	*CKAN_02314700*	0.19675	0.529168	0.37181
CcbHLH131	*CKAN_02520600*	0.059031	0.103492	0.570396
CcbHLH132	*CKAN_02513000*	0.01326	0.035232	0.376368
CcbHLH133	*CKAN_02472300*	0.003183	0.034546	0.09214
CcbHLH134	*CKAN_02471500*	0.010231	0.028222	0.362517
CcbHLH135	*CKAN_02470600*	0.013687	0.020229	0.676603
CcbHLH136	*CKAN_02457600*	0.004965	0.042615	0.116505
CcbHLH137	*CKAN_02447100*	0.030267	0.033274	0.909613
CcbHLH139	*CKAN_02430000*	0.015287	0.028677	0.533075
CcbHLH140	*CKAN_02428500*	0.083907	0.149307	0.561978
CcbHLH141	*CKAN_02413200*	0.007863	0.035353	0.222409
CcbHLH142	*CKAN_02405400*	0.002505	0.034801	0.071979
CcbHLH144	*CKAN_01263000*	0.113423	0.332149	0.341483
CcbHLH146	*CKAN_01538500*	0.180344	0.831961	0.21677
CcbHLH149	*CKAN_01813000*	0.251195	1.063736	0.236144
CcbHLH145	*CKAN_02255000*	0.228829	1.624656	0.140848
CcbHLH144	*CKAN_02621200*	0.016539	0.020409	0.810347
CcbHLH145	*CKAN_02582300*	0.001432	0.021749	0.065832
CcbHLH146	*CKAN_02573200*	0.013522	0.048354	0.279654
CcbHLH148	*CKAN_02553800*	0.029111	0.07446	0.390964
CcbHLH150	*CKAN_02543500*	0.002565	0.022286	0.115089
CcbHLH147	*CKAN_02685100*	0.274031	0.651972	0.420311
CcbHLH028	*CKAN_00210400*	0.235583	0.887033	0.265585
CcbHLH027	*CKAN_00086200*	0.200663	1.316786	0.152389
CcbHLH028	*CKAN_00093200*	0.290669	1.030825	0.281977
CcbHLH036	*CKAN_00083300*	0.424642	1.131448	0.375308
CcbHLH031	*CKAN_00238300*	0.206419	1.0341	0.199613
CcbHLH033	*CKAN_00235600*	0.15278	0.962703	0.158698
CcbHLH019	*CKAN_00384000*	0.011908	0.044579	0.267115
CcbHLH020	*CKAN_00486600*	0.088955	0.167251	0.531867
CcbHLH021	*CKAN_00510700*	0.007166	0.024933	0.287396
CcbHLH022	*CKAN_00511800*	0.010954	0.034282	0.319535

CcbHLH023	*CKAN_00514000*	0.015813	0.041657	0.379614
CcbHLH024	*CKAN_00528300*	0.006891	0.034924	0.197305
CcbHLH025	*CKAN_00549500*	0.014469	0.042322	0.341869
CcbHLH026	*CKAN_00605500*	0.046828	0.047088	0.994463
CcbHLH027	*CKAN_00615000*	0.015715	0.053334	0.294649
CcbHLH028	*CKAN_00628900*	0.001959	0.034351	0.057035
CcbHLH029	*CKAN_00635900*	0.008784	0.014693	0.597847
CcbHLH030	*CKAN_00638100*	0.011378	0.004323	2.632154
CcbHLH031	*CKAN_00639100*	0.001303	0.019049	0.068385
CcbHLH032	*CKAN_00640000*	0.002922	0.05452	0.053592
CcbHLH033	*CKAN_00640400*	0.001185	0.03304	0.035867
CcbHLH034	*CKAN_00643700*	0.060853	0.15026	0.404985
CcbHLH035	*CKAN_00649400*	0.015702	0	#NAME?
CcbHLH036	*CKAN_00657400*	0.008501	0.019267	0.441222
CcbHLH037	*CKAN_00661800*	0.01844	0.051578	0.357514
CcbHLH018	*CKAN_00360900*	0.001674	0	#NAME?
CcbHLH029	*CKAN_00640000*	0.572202	NaN	NaN
CcbHLH032	*CKAN_00635900*	0.605557	NaN	NaN
CcbHLH028	*CKAN_01157500*	0.189233	0.539707	0.350621
CcbHLH029	*CKAN_01161300*	0.22523	1.054793	0.21353
CcbHLH031	*CKAN_01163000*	0.143667	0.59202	0.242672
CcbHLH033	*CKAN_01163500*	0.174111	0.501592	0.347117
CcbHLH035	*CKAN_01167200*	0.213437	0.541122	0.394434
CcbHLH027	*CKAN_01152000*	0.107629	0.680555	0.158148
CcbHLH036	*CKAN_01149700*	0.577273	1.463614	0.394416
CcbHLH018	*CKAN_01137600*	0.092781	0.949391	0.097727
CcbHLH025	*CKAN_01382600*	0.255355	1.312404	0.19457
CcbHLH018	*CKAN_02065000*	0.197107	1.407793	0.140011
CcbHLH019	*CKAN_02028400*	0.227486	1.197159	0.190022
CcbHLH021	*CKAN_02470800*	0.140858	0.471001	0.299061
CcbHLH022	*CKAN_02471500*	0.130382	0.726845	0.17938
CcbHLH023	*CKAN_02472300*	0.141022	1.167334	0.120807
CcbHLH020	*CKAN_02457600*	0.319308	0.668907	0.477357
CcbHLH041	*CKAN_00297600*	0.130301	0.665314	0.19585
CcbHLH038	*CKAN_00692700*	0.005147	0.039895	0.129019
CcbHLH039	*CKAN_00742100*	0.004215	0.020113	0.209565
CcbHLH040	*CKAN_00844100*	0.022616	0.033491	0.675281
CcbHLH041	*CKAN_00851700*	0.015263	0.010268	1.486515
CcbHLH043	*CKAN_00959300*	0.039983	0.065873	0.606978
CcbHLH044	*CKAN_00968000*	0.006578	0.032895	0.199957
CcbHLH045	*CKAN_00968800*	0.025345	0.048016	0.527839
CcbHLH046	*CKAN_00973600*	0.022574	0.025457	0.886761
CcbHLH047	*CKAN_01002200*	0	0	NaN

CcbHLH043	*CKAN_01683800*	0.361091	0.728616	0.495585
CcbHLH045	*CKAN_01571900*	0.255622	1.727308	0.147988
CcbHLH043	*CKAN_01548800*	0.406237	1.383142	0.293706
CcbHLH046	*CKAN_01620800*	0.269822	0.580204	0.465047
CcbHLH041	*CKAN_01610300*	0.319489	1.40791	0.226925
CcbHLH043	*CKAN_01847800*	0.402001	1.299401	0.309374
CcbHLH051	*CKAN_00278200*	0.014097	0.054514	0.258598
CcbHLH055	*CKAN_00235600*	0.270347	1.02085	0.264826
CcbHLH056	*CKAN_00238300*	0.265179	0.925431	0.286546
CcbHLH058	*CKAN_00093200*	0.355491	0.94915	0.374536
CcbHLH059	*CKAN_00086200*	0.227377	1.165972	0.19501
CcbHLH060	*CKAN_00083300*	0.494321	1.538377	0.321326
CcbHLH058	*CKAN_00210400*	0.291649	1.012303	0.288104
CcbHLH060	*CKAN_00657400*	0.286115	0.780505	0.366576
CcbHLH061	*CKAN_00360900*	0.094691	0.926769	0.102173
CcbHLH062	*CKAN_00649400*	0.091712	0.736794	0.124474
CcbHLH054	*CKAN_00649400*	0.085124	0.427015	0.199347
CcbHLH055	*CKAN_00640400*	0.174708	0.557897	0.313156
CcbHLH056	*CKAN_00639100*	0.144982	0.576776	0.251366
CcbHLH057	*CKAN_00635900*	0.2384	1.019443	0.233853
CcbHLH058	*CKAN_00628900*	0.192513	0.498978	0.385814
CcbHLH059	*CKAN_00615000*	0.109085	0.613379	0.177842
CcbHLH062	*CKAN_01167200*	0.313305	1.171819	0.267366
CcbHLH052	*CKAN_01216400*	0.012862	0.010209	1.259926
CcbHLH053	*CKAN_01202000*	0.0263	0.037144	0.708041
CcbHLH055	*CKAN_01163500*	0.008355	0.02082	0.401291
CcbHLH056	*CKAN_01163000*	0.003944	0.019641	0.2008
CcbHLH057	*CKAN_01161300*	0.004445	0.027258	0.163082
CcbHLH058	*CKAN_01157500*	0.035265	0.039626	0.88994
CcbHLH059	*CKAN_01152000*	0.007901	0.054029	0.146238
CcbHLH061	*CKAN_01137600*	0.003301	0.012091	0.272997
CcbHLH063	*CKAN_01116500*	0.004192	0.013865	0.30235
CcbHLH064	*CKAN_01112300*	0.026598	0.047394	0.5612
CcbHLH065	*CKAN_01094000*	0.016096	0.02629	0.612256
CcbHLH067	*CKAN_01070800*	0.147698	0.277406	0.532427
CcbHLH049	*CKAN_01263000*	0	0.031751	0
CcbHLH051	*CKAN_01238400*	0.003495	0.011829	0.295466
CcbHLH067	*CKAN_01446500*	0.172181	0.525697	0.327529
CcbHLH061	*CKAN_02065000*	0.197359	1.219051	0.161896
CcbHLH065	*CKAN_02314700*	0.316309	0.916714	0.345047
CcbHLH049	*CKAN_02621200*	0.11089	0.34233	0.323926
CcbHLH048	*CKAN_02685100*	0.004277	0.008043	0.531714
CcbHLH075	*CKAN_00549500*	0.273117	1.18865	0.229771
CcbHLH076	*CKAN_01070800*	0.160301	0.54792	0.292562

CcbHLH076	*CKAN_01446500*	0.004912	0.008863	0.554232
CcbHLH078	*CKAN_01464100*	0.004464	0.025567	0.17459
CcbHLH079	*CKAN_01466700*	0.010903	0.0125	0.872242
CcbHLH070	*CKAN_01298000*	0.003806	0.027494	0.138424
CcbHLH074	*CKAN_01381200*	0.001555	0.036405	0.042726
CcbHLH075	*CKAN_01382600*	0.016822	0.03752	0.448343
CcbHLH079	*CKAN_01773700*	0.28431	0.918134	0.30966
CcbHLH070	*CKAN_02204900*	0.23415	0.935347	0.250335
CcbHLH076	*CKAN_02323400*	0.334966	0.843777	0.396983
CcbHLH082	*CKAN_00138900*	0.152852	0.71575	0.213555
CcbHLH082	*CKAN_00278200*	0.194575	1.063363	0.182981
CcbHLH091	*CKAN_00973600*	0.27911	0.554032	0.503781
CcbHLH080	*CKAN_00851700*	0.315381	1.38842	0.227151
CcbHLH087	*CKAN_00959300*	0.356791	0.641412	0.55626
CcbHLH089	*CKAN_00929600*	0.218791	0.95217	0.229781
CcbHLH083	*CKAN_00968800*	0.270879	1.841344	0.147109
CcbHLH084	*CKAN_00959300*	0.419967	1.546067	0.271636
CcbHLH090	*CKAN_01571900*	0.201567	2.370151	0.085044
CcbHLH083	*CKAN_01626400*	0.196065	2.238392	0.087592
CcbHLH080	*CKAN_01610300*	0.015603	0.014926	1.045343
CcbHLH081	*CKAN_01588000*	0.002447	0.008811	0.27773
CcbHLH082	*CKAN_01585700*	0.000849	0.014635	0.05799
CcbHLH083	*CKAN_01571900*	0.004101	0.025021	0.163907
CcbHLH084	*CKAN_01548800*	0.038521	0.059955	0.6425
CcbHLH085	*CKAN_01538500*	0.001698	0.044706	0.037977
CcbHLH086	*CKAN_01524900*	0.044269	0.05209	0.849842
CcbHLH087	*CKAN_01683800*	0.073755	0.16527	0.446272
CcbHLH088	*CKAN_01666800*	0.005409	0.023146	0.233688
CcbHLH089	*CKAN_01640400*	0.007676	0.07642	0.100445
CcbHLH090	*CKAN_01626400*	0.002635	0.028295	0.093118
CcbHLH091	*CKAN_01620700*	0.029413	0.050826	0.578707
CcbHLH087	*CKAN_01548800*	0.417748	1.606017	0.260114
CcbHLH084	*CKAN_01683800*	0.447875	1.302331	0.343903
CcbHLH084	*CKAN_01847800*	0.199015	0.875915	0.227209
CcbHLH088	*CKAN_01859800*	0.237518	1.253209	0.189528
CcbHLH087	*CKAN_01847800*	0.439215	1.259685	0.348671
CcbHLH089	*CKAN_01887300*	0.296173	1.227583	0.241265
CcbHLH085	*CKAN_02573200*	0.193504	0.888961	0.217675
CcbHLH094	*CKAN_00959300*	0.446755	1.251257	0.357045
CcbHLH092	*CKAN_00929600*	0.366609	1.030485	0.355763
CcbHLH101	*CKAN_01466700*	0.267962	0.867458	0.308905
CcbHLH094	*CKAN_01548800*	0.239988	0.846223	0.283599
CcbHLH093	*CKAN_01666800*	0.231278	1.198621	0.192953

CcbHLH094	*CKAN_01683800*	0.496511	1.237931	0.401081
CcbHLH092	*CKAN_01640400*	0.29541	1.220625	0.242015
CcbHLH092	*CKAN_01887300*	0.023351	0.037731	0.618876
CcbHLH093	*CKAN_01859800*	0.004584	0.023562	0.194535
CcbHLH094	*CKAN_01847800*	0.0032	0.022101	0.14479
CcbHLH096	*CKAN_01845800*	0.011973	0.024392	0.490845
CcbHLH097	*CKAN_01813000*	0.002374	0.013349	0.177874
CcbHLH099	*CKAN_01794000*	0.001834	0.021128	0.086791
CcbHLH100	*CKAN_01790000*	0.004336	0.01806	0.240112
CcbHLH101	*CKAN_01773700*	0.011389	0.025081	0.45408
CcbHLH102	*CKAN_01718100*	0.023189	0.073473	0.315619
CcbHLH108	*CKAN_00384000*	0.292859	1.338536	0.21879
CcbHLH109	*CKAN_00360900*	0.178678	1.482109	0.120557
CcbHLH109	*CKAN_01137600*	0.190645	1.218496	0.156459
CcbHLH103	*CKAN_01288800*	0.114829	0.638054	0.179968
CcbHLH105	*CKAN_01347400*	0.109519	0.563968	0.194193
CcbHLH103	*CKAN_01903400*	0.002757	0.023276	0.118438
CcbHLH104	*CKAN_01917400*	0.004105	0.006953	0.590486
CcbHLH105	*CKAN_01956500*	0.003746	0.006487	0.577581
CcbHLH106	*CKAN_01959800*	0.003372	0.021375	0.157761
CcbHLH107	*CKAN_02009900*	0.008532	0.006414	1.330311
CcbHLH108	*CKAN_02028400*	0.001034	0.022668	0.045629
CcbHLH109	*CKAN_02065000*	0.009475	0.01821	0.520313
CcbHLH103	*CKAN_02168200*	0.1902	1.167879	0.162859
CcbHLH106	*CKAN_02212300*	0.234394	0.888547	0.263795
CcbHLH104	*CKAN_02178900*	0.184425	1.328595	0.138812
CcbHLH112	*CKAN_01298000*	0.235369	1.046345	0.224944
CcbHLH115	*CKAN_01288800*	0.180647	1.014643	0.178041
CcbHLH115	*CKAN_01903400*	0.192819	1.15363	0.167141
CcbHLH111	*CKAN_01959800*	0.222376	0.952893	0.23337
CcbHLH113	*CKAN_01917400*	0.170995	1.309259	0.130605
CcbHLH111	*CKAN_02212300*	0.002764	0.015654	0.176589
CcbHLH112	*CKAN_02204900*	0.007521	0.019545	0.384814
CcbHLH113	*CKAN_02178900*	0.002011	0.020272	0.09919
CcbHLH114	*CKAN_02171400*	0.005622	0.035009	0.160599
CcbHLH115	*CKAN_02168200*	0.004112	0.013986	0.294029
CcbHLH116	*CKAN_02156200*	0.00755	0.03025	0.249596
CcbHLH117	*CKAN_02150200*	0.089641	0.128287	0.698757
CcbHLH118	*CKAN_02091900*	0.011999	0.010222	1.173874
CcbHLH119	*CKAN_02081300*	0.004198	0.012481	0.336333
CcbHLH120	*CKAN_02081200*	0.006048	0.043808	0.138066

Table S6. *CcbHLHs* protein interaction analysis

Cinnamomun camphcra protein	*Cinnamomun camphcra* protein	*Arabidopsis thaliana* protein	*Arabidopsis thaliana* protein	score
CcbHLH005	CcbHLH148	AT5G62610.1	AT1G29950.2	0.618
CcbHLH005	CcbHLH110	AT5G62610.1	AT1G31050.1	0.576
CcbHLH005	CcbHLH146	AT5G62610.1	AT1G68810.1	0.59
CcbHLH005	CcbHLH144	AT5G62610.1	AT2G31730.1	0.485
CcbHLH005	CcbHLH016	AT5G62610.1	AT2G41130.1	0.619
CcbHLH005	CcbHLH117	AT5G62610.1	AT3G19860.2	0.402
CcbHLH005	CcbHLH112	AT5G62610.1	AT3G20640.1	0.637
CcbHLH005	CcbHLH038	AT5G62610.1	AT3G47640.2	0.599
CcbHLH005	CcbHLH072	AT5G62610.1	AT4G16430.1	0.615
CcbHLH005	CcbHLH030	AT5G62610.1	AT4G29100.1	0.605
CcbHLH005	CcbHLH043	AT5G62610.1	AT5G51780.1	0.616
CcbHLH005	CcbHLH122	AT5G62610.1	AT5G53210.1	0.485
CcbHLH005	CcbHLH036	AT5G62610.1	AT5G56960.1	0.72
CcbHLH005	CcbHLH056	AT5G62610.1	AT5G65640.1	0.461
CcbHLH007	CcbHLH137	AT5G61270.1	AT1G09530.2	0.635
CcbHLH007	CcbHLH150	AT5G61270.1	AT2G20180.2	0.641
CcbHLH007	CcbHLH079	AT5G61270.1	AT2G43010.1	0.704
CcbHLH007	CcbHLH138	AT5G61270.1	AT3G06120.1	0.424
CcbHLH007	CcbHLH074	AT5G61270.1	AT5G08130.5	0.449
CcbHLH011	CcbHLH148	AT2G42300.1	AT1G29950.2	0.616
CcbHLH011	CcbHLH146	AT2G42300.1	AT1G68810.1	0.584
CcbHLH011	CcbHLH016	AT2G42300.1	AT2G41130.1	0.6
CcbHLH011	CcbHLH038	AT2G42300.1	AT3G47640.2	0.416
CcbHLH011	CcbHLH072	AT2G42300.1	AT4G16430.1	0.598
CcbHLH011	CcbHLH030	AT2G42300.1	AT4G29100.1	0.604
CcbHLH011	CcbHLH043	AT2G42300.1	AT5G51780.1	0.615
CcbHLH011	CcbHLH056	AT2G42300.1	AT5G65640.1	0.626
CcbHLH011	CcbHLH112	AT2G42300.1	AT3G20640.1	0.636
CcbHLH011	CcbHLH036	AT2G42300.1	AT5G56960.1	0.695
CcbHLH016	CcbHLH148	AT2G41130.1	AT1G29950.2	0.615
CcbHLH016	CcbHLH110	AT2G41130.1	AT1G31050.1	0.416
CcbHLH016	CcbHLH117	AT2G41130.1	AT3G19860.2	0.402
CcbHLH016	CcbHLH096	AT2G41130.1	AT5G43650.1	0.416
CcbHLH016	CcbHLH056	AT2G41130.1	AT5G65640.1	0.448
CcbHLH016	CcbHLH064	AT2G41130.1	AT4G34530.1	0.457
CcbHLH016	CcbHLH109	AT2G41130.1	AT5G57150.4	0.468
CcbHLH016	CcbHLH038	AT2G41130.1	AT3G47640.2	0.491
CcbHLH016	CcbHLH081	AT2G41130.1	AT5G50915.1	0.551
CcbHLH016	CcbHLH030	AT2G41130.1	AT4G29100.1	0.592
CcbHLH016	CcbHLH043	AT2G41130.1	AT5G51780.1	0.597
CcbHLH016	CcbHLH112	AT2G41130.1	AT3G20640.1	0.632
CcbHLH016	CcbHLH036	AT2G41130.1	AT5G56960.1	0.683

CcbHLH024	CcbHLH050	AT3G19500.1	AT1G49770.1	0.45
CcbHLH024	CcbHLH139	AT3G19500.1	AT1G68920.1	0.592
CcbHLH024	CcbHLH091	AT3G19500.1	AT2G31210.1	0.581
CcbHLH024	CcbHLH105	AT3G19500.1	AT5G54680.1	0.441
CcbHLH024	CcbHLH081	AT3G19500.1	AT5G50915.1	0.518
CcbHLH024	CcbHLH109	AT3G19500.1	AT5G57150.4	0.567
CcbHLH024	CcbHLH092	AT3G19500.1	AT4G00050.1	0.616
CcbHLH024	CcbHLH055	AT3G19500.1	AT4G37850.1	0.636
CcbHLH026	CcbHLH144	AT4G36930.1	AT2G31730.1	0.487
CcbHLH026	CcbHLH113	AT4G36930.1	AT3G50330.1	0.616
CcbHLH026	CcbHLH036	AT4G36930.1	AT5G56960.1	0.59
CcbHLH026	CcbHLH111	AT4G36930.1	AT5G09750.1	0.596
CcbHLH030	CcbHLH148	AT4G29100.1	AT1G29950.2	0.597
CcbHLH030	CcbHLH146	AT4G29100.1	AT1G68810.1	0.645
CcbHLH030	CcbHLH117	AT4G29100.1	AT3G19860.2	0.402
CcbHLH030	CcbHLH038	AT4G29100.1	AT3G47640.2	0.459
CcbHLH030	CcbHLH056	AT4G29100.1	AT5G65640.1	0.417
CcbHLH030	CcbHLH064	AT4G29100.1	AT4G34530.1	0.48
CcbHLH030	CcbHLH043	AT4G29100.1	AT5G51780.1	0.586
CcbHLH030	CcbHLH036	AT4G29100.1	AT5G56960.1	0.639
CcbHLH030	CcbHLH081	AT4G29100.1	AT5G50915.1	0.658
CcbHLH036	CcbHLH148	AT5G56960.1	AT1G29950.2	0.709
CcbHLH036	CcbHLH050	AT5G56960.1	AT1G49770.1	0.618
CcbHLH036	CcbHLH146	AT5G56960.1	AT1G68810.1	0.743
CcbHLH036	CcbHLH052	AT5G56960.1	AT2G28160.1	0.628
CcbHLH036	CcbHLH095	AT5G56960.1	AT2G31220.1	0.753
CcbHLH036	CcbHLH112	AT5G56960.1	AT3G20640.1	0.803
CcbHLH036	CcbHLH038	AT5G56960.1	AT3G47640.2	0.548
CcbHLH036	CcbHLH081	AT5G56960.1	AT5G50915.1	0.633
CcbHLH036	CcbHLH043	AT5G56960.1	AT5G51780.1	0.686
CcbHLH036	CcbHLH056	AT5G56960.1	AT5G65640.1	0.6
CcbHLH038	CcbHLH115	AT3G47640.2	AT1G22490.1	0.514
CcbHLH038	CcbHLH148	AT3G47640.2	AT1G29950.2	0.416
CcbHLH038	CcbHLH110	AT3G47640.2	AT1G31050.1	0.609
CcbHLH038	CcbHLH059	AT3G47640.2	AT1G59640.2	0.473
CcbHLH038	CcbHLH052	AT3G47640.2	AT2G28160.1	0.797
CcbHLH038	CcbHLH144	AT3G47640.2	AT2G31730.1	0.598
CcbHLH038	CcbHLH138	AT3G47640.2	AT3G06120.1	0.687
CcbHLH038	CcbHLH080	AT3G47640.2	AT3G07340.1	0.517
CcbHLH038	CcbHLH112	AT3G47640.2	AT3G20640.1	0.434
CcbHLH038	CcbHLH064	AT3G47640.2	AT4G34530.1	0.559
CcbHLH038	CcbHLH056	AT3G47640.2	AT5G65640.1	0.679
CcbHLH038	CcbHLH092	AT3G47640.2	AT4G00050.1	0.682
CcbHLH038	CcbHLH122	AT3G47640.2	AT5G53210.1	0.738
CcbHLH038	CcbHLH120	AT3G47640.2	AT4G14410.1	0.747
CcbHLH038	CcbHLH081	AT3G47640.2	AT5G50915.1	0.784

CcbHLH038	CcbHLH100	AT3G47640.2	AT4G02590.1	0.787
CcbHLH038	CcbHLH105	AT3G47640.2	AT5G54680.1	0.863
CcbHLH038	CcbHLH132	AT3G47640.2	AT3G56980.1	0.953
CcbHLH038	CcbHLH040	AT3G47640.2	AT3G56970.1	0.955
CcbHLH040	CcbHLH115	AT3G56970.1	AT1G22490.1	0.532
CcbHLH040	CcbHLH110	AT3G56970.1	AT1G31050.1	0.483
CcbHLH040	CcbHLH052	AT3G56970.1	AT2G28160.1	0.977
CcbHLH040	CcbHLH092	AT3G56970.1	AT4G00050.1	0.453
CcbHLH040	CcbHLH132	AT3G56970.1	AT3G56980.1	0.498
CcbHLH040	CcbHLH081	AT3G56970.1	AT5G50915.1	0.591
CcbHLH040	CcbHLH055	AT3G56970.1	AT4G37850.1	0.594
CcbHLH040	CcbHLH120	AT3G56970.1	AT4G14410.1	0.914
CcbHLH040	CcbHLH105	AT3G56970.1	AT5G54680.1	0.929
CcbHLH043	CcbHLH115	AT5G51780.1	AT1G22490.1	0.659
CcbHLH043	CcbHLH148	AT5G51780.1	AT1G29950.2	0.616
CcbHLH043	CcbHLH146	AT5G51780.1	AT1G68810.1	0.581
CcbHLH043	CcbHLH112	AT5G51780.1	AT3G20640.1	0.634
CcbHLH043	CcbHLH093	AT5G51780.1	AT3G61950.1	0.65
CcbHLH043	CcbHLH081	AT5G51780.1	AT5G50915.1	0.601
CcbHLH043	CcbHLH056	AT5G51780.1	AT5G65640.1	0.429
CcbHLH045	CcbHLH099	AT4G17880.1	AT1G32640.1	0.546
CcbHLH045	CcbHLH081	AT4G17880.1	AT5G50915.1	0.626
CcbHLH046	CcbHLH109	AT1G06170.2	AT5G57150.4	0.402
CcbHLH046	CcbHLH116	AT1G06170.2	AT2G16910.1	0.43
CcbHLH046	CcbHLH108	AT1G06170.2	AT2G24260.1	0.484
CcbHLH046	CcbHLH146	AT1G06170.2	AT1G68810.1	0.506
CcbHLH046	CcbHLH096	AT1G06170.2	AT5G43650.1	0.516
CcbHLH046	CcbHLH136	AT1G06170.2	AT1G09250.1	0.629
CcbHLH050	CcbHLH105	AT1G49770.1	AT5G54680.1	0.461
CcbHLH050	CcbHLH095	AT1G49770.1	AT2G31220.1	0.545
CcbHLH050	CcbHLH054	AT1G49770.1	AT4G33880.1	0.679
CcbHLH050	CcbHLH062	AT1G49770.1	AT2G14760.3	0.679
CcbHLH050	CcbHLH082	AT1G49770.1	AT3G26744.1	0.913
CcbHLH052	CcbHLH063	AT2G28160.1	AT1G27660.1	0.749
CcbHLH052	CcbHLH120	AT2G28160.1	AT4G14410.1	0.637
CcbHLH052	CcbHLH105	AT2G28160.1	AT5G54680.1	0.68
CcbHLH052	CcbHLH132	AT2G28160.1	AT3G56980.1	0.959
CcbHLH054	CcbHLH134	AT4G33880.1	AT1G63650.3	0.548
CcbHLH054	CcbHLH077	AT4G33880.1	AT1G66470.1	0.568
CcbHLH054	CcbHLH134	AT4G33880.1	AT4G00480.2	0.429
CcbHLH054	CcbHLH094	AT4G33880.1	AT5G51790.1	0.52
CcbHLH054	CcbHLH134	AT4G33880.1	AT5G41315.1	0.581
CcbHLH054	CcbHLH131	AT4G33880.1	AT5G58010.1	0.592
CcbHLH055	CcbHLH110	AT4G37850.1	AT1G31050.1	0.62
CcbHLH055	CcbHLH146	AT4G37850.1	AT1G68810.1	0.465
CcbHLH055	CcbHLH139	AT4G37850.1	AT1G68920.1	0.568

CcbHLH055	CcbHLH112	AT4G37850.1	AT3G20640.1	0.548
CcbHLH055	CcbHLH132	AT4G37850.1	AT3G56980.1	0.463
CcbHLH055	CcbHLH100	AT4G37850.1	AT4G02590.1	0.465
CcbHLH055	CcbHLH120	AT4G37850.1	AT4G14410.1	0.463
CcbHLH055	CcbHLH064	AT4G37850.1	AT4G34530.1	0.597
CcbHLH055	CcbHLH105	AT4G37850.1	AT5G54680.1	0.441
CcbHLH056	CcbHLH148	AT5G65640.1	AT1G29950.2	0.564
CcbHLH056	CcbHLH141	AT5G65640.1	AT1G73830.1	0.506
CcbHLH056	CcbHLH058	AT5G65640.1	AT2G42280.1	0.615
CcbHLH056	CcbHLH138	AT5G65640.1	AT3G06120.1	0.548
CcbHLH056	CcbHLH112	AT5G65640.1	AT3G20640.1	0.52
CcbHLH056	CcbHLH142	AT5G65640.1	AT3G24140.1	0.831
CcbHLH056	CcbHLH081	AT5G65640.1	AT5G50915.1	0.569
CcbHLH056	CcbHLH122	AT5G65640.1	AT5G53210.1	0.447
CcbHLH059	CcbHLH119	AT1G59640.2	AT1G06150.1	0.463
CcbHLH059	CcbHLH110	AT1G59640.2	AT1G31050.1	0.576
CcbHLH059	CcbHLH117	AT1G59640.2	AT3G19860.2	0.402
CcbHLH059	CcbHLH149	AT1G59640.2	AT3G06590.2	0.467
CcbHLH059	CcbHLH144	AT1G59640.2	AT2G31730.1	0.485
CcbHLH059	CcbHLH122	AT1G59640.2	AT5G53210.1	0.485
CcbHLH062	CcbHLH146	AT2G14760.3	AT1G68810.1	0.541
CcbHLH062	CcbHLH134	AT2G14760.3	AT5G41315.1	0.452
CcbHLH062	CcbHLH105	AT2G14760.3	AT5G54680.1	0.519
CcbHLH062	CcbHLH134	AT2G14760.3	AT4G00480.2	0.564
CcbHLH064	CcbHLH149	AT4G34530.1	AT3G06590.2	0.5
CcbHLH064	CcbHLH080	AT4G34530.1	AT3G07340.1	0.573
CcbHLH064	CcbHLH117	AT4G34530.1	AT3G19860.2	0.515
CcbHLH064	CcbHLH134	AT4G34530.1	AT5G41315.1	0.524
CcbHLH064	CcbHLH122	AT4G34530.1	AT5G53210.1	0.562
CcbHLH072	CcbHLH098	AT4G16430.1	AT2G34820.1	0.446
CcbHLH072	CcbHLH074	AT4G16430.1	AT5G08130.5	0.441
CcbHLH074	CcbHLH139	AT5G08130.5	AT1G68920.1	0.519
CcbHLH074	CcbHLH140	AT5G08130.5	AT1G69010.1	0.57
CcbHLH074	CcbHLH141	AT5G08130.5	AT1G73830.1	0.593
CcbHLH074	CcbHLH095	AT5G08130.5	AT2G31220.1	0.461
CcbHLH074	CcbHLH079	AT5G08130.5	AT2G43010.1	0.616
CcbHLH074	CcbHLH105	AT5G08130.5	AT5G54680.1	0.441
CcbHLH077	CcbHLH110	AT1G66470.1	AT1G31050.1	0.501
CcbHLH077	CcbHLH134	AT1G66470.1	AT1G63650.3	0.63
CcbHLH077	CcbHLH108	AT1G66470.1	AT2G24260.1	0.405
CcbHLH077	CcbHLH096	AT1G66470.1	AT5G43650.1	0.483
CcbHLH077	CcbHLH134	AT1G66470.1	AT4G00480.2	0.54
CcbHLH077	CcbHLH131	AT1G66470.1	AT5G58010.1	0.607
CcbHLH077	CcbHLH134	AT1G66470.1	AT5G41315.1	0.62
CcbHLH079	CcbHLH137	AT2G43010.1	AT1G09530.2	0.965
CcbHLH079	CcbHLH150	AT2G43010.1	AT2G20180.2	0.664

CcbHLH079	CcbHLH122	AT2G43010.1	AT5G53210.1	0.428
CcbHLH080	CcbHLH110	AT3G07340.1	AT1G31050.1	0.515
CcbHLH080	CcbHLH144	AT3G07340.1	AT2G31730.1	0.4
CcbHLH080	CcbHLH122	AT3G07340.1	AT5G53210.1	0.407
CcbHLH080	CcbHLH128	AT3G07340.1	AT4G20970.1	0.651
CcbHLH081	CcbHLH126	AT5G50915.1	AT1G10586.1	0.624
CcbHLH081	CcbHLH115	AT5G50915.1	AT1G22490.1	0.514
CcbHLH081	CcbHLH148	AT5G50915.1	AT1G29950.2	0.545
CcbHLH081	CcbHLH110	AT5G50915.1	AT1G31050.1	0.609
CcbHLH081	CcbHLH099	AT5G50915.1	AT1G32640.1	0.425
CcbHLH081	CcbHLH146	AT5G50915.1	AT1G68810.1	0.565
CcbHLH081	CcbHLH144	AT5G50915.1	AT2G31730.1	0.576
CcbHLH081	CcbHLH138	AT5G50915.1	AT3G06120.1	0.72
CcbHLH081	CcbHLH112	AT5G50915.1	AT3G20640.1	0.721
CcbHLH081	CcbHLH082	AT5G50915.1	AT3G26744.1	0.622
CcbHLH081	CcbHLH132	AT5G50915.1	AT3G56980.1	0.591
CcbHLH081	CcbHLH134	AT5G50915.1	AT4G00480.2	0.686
CcbHLH081	CcbHLH134	AT5G50915.1	AT4G09820.1	0.494
CcbHLH081	CcbHLH134	AT5G50915.1	AT5G41315.1	0.565
CcbHLH081	CcbHLH122	AT5G50915.1	AT5G53210.1	0.597
CcbHLH082	CcbHLH138	AT3G26744.1	AT3G06120.1	0.966
CcbHLH082	CcbHLH142	AT3G26744.1	AT3G24140.1	0.722
CcbHLH082	CcbHLH093	AT3G26744.1	AT3G61950.1	0.449
CcbHLH082	CcbHLH105	AT3G26744.1	AT5G54680.1	0.55
CcbHLH082	CcbHLH122	AT3G26744.1	AT5G53210.1	0.983
CcbHLH091	CcbHLH146	AT2G31210.1	AT1G68810.1	0.551
CcbHLH091	CcbHLH116	AT2G31210.1	AT2G16910.1	0.439
CcbHLH091	CcbHLH108	AT2G31210.1	AT2G24260.1	0.518
CcbHLH091	CcbHLH109	AT2G31210.1	AT5G57150.4	0.431
CcbHLH091	CcbHLH096	AT2G31210.1	AT5G43650.1	0.535
CcbHLH092	CcbHLH119	AT4G00050.1	AT1G06150.1	0.594
CcbHLH092	CcbHLH138	AT4G00050.1	AT3G06120.1	0.432
CcbHLH092	CcbHLH105	AT4G00050.1	AT5G54680.1	0.416
CcbHLH092	CcbHLH122	AT4G00050.1	AT5G53210.1	0.66
CcbHLH093	CcbHLH145	AT3G61950.1	AT1G01260.1	0.519
CcbHLH093	CcbHLH139	AT3G61950.1	AT1G68920.1	0.582
CcbHLH093	CcbHLH109	AT3G61950.1	AT5G57150.4	0.553
CcbHLH095	CcbHLH146	AT2G31220.1	AT1G68810.1	0.621
CcbHLH095	CcbHLH116	AT2G31220.1	AT2G16910.1	0.429
CcbHLH095	CcbHLH109	AT2G31220.1	AT5G57150.4	0.402
CcbHLH095	CcbHLH096	AT2G31220.1	AT5G43650.1	0.516
CcbHLH096	CcbHLH115	AT5G43650.1	AT1G22490.1	0.514
CcbHLH096	CcbHLH109	AT5G43650.1	AT5G57150.4	0.963
CcbHLH100	CcbHLH110	AT4G02590.1	AT1G31050.1	0.79
CcbHLH100	CcbHLH105	AT4G02590.1	AT5G54680.1	0.542
CcbHLH105	CcbHLH140	AT5G54680.1	AT1G69010.1	0.451

CcbHLH105	CcbHLH132	AT5G54680.1	AT3G56980.1	0.871
CcbHLH105	CcbHLH120	AT5G54680.1	AT4G14410.1	0.558
CcbHLH108	CcbHLH134	AT2G24260.1	AT4G00480.2	0.459
CcbHLH109	CcbHLH110	AT5G57150.4	AT1G31050.1	0.618
CcbHLH109	CcbHLH139	AT5G57150.4	AT1G68920.1	0.55
CcbHLH109	CcbHLH140	AT5G57150.4	AT1G69010.1	0.556
CcbHLH109	CcbHLH131	AT5G57150.4	AT5G58010.1	0.515
CcbHLH110	CcbHLH115	AT1G31050.1	AT1G22490.1	0.514
CcbHLH110	CcbHLH132	AT1G31050.1	AT3G56980.1	0.513
CcbHLH110	CcbHLH122	AT1G31050.1	AT5G53210.1	0.605
CcbHLH110	CcbHLH146	AT1G31050.1	AT1G68810.1	0.643
CcbHLH112	CcbHLH148	AT3G20640.1	AT1G29950.2	0.637
CcbHLH112	CcbHLH146	AT3G20640.1	AT1G68810.1	0.637
CcbHLH112	CcbHLH139	AT3G20640.1	AT1G68920.1	0.581
CcbHLH119	CcbHLH139	AT1G06150.1	AT1G68920.1	0.549
CcbHLH120	CcbHLH132	AT4G14410.1	AT3G56980.1	0.872
CcbHLH122	CcbHLH137	AT5G53210.1	AT1G09530.2	0.447
CcbHLH122	CcbHLH144	AT5G53210.1	AT2G31730.1	0.485
CcbHLH122	CcbHLH132	AT5G53210.1	AT3G56980.1	0.415
CcbHLH122	CcbHLH128	AT5G53210.1	AT4G20970.1	0.507
CcbHLH126	CcbHLH134	AT1G10586.1	AT4G09820.1	0.546
CcbHLH137	CcbHLH138	AT1G09530.2	AT3G06120.1	0.43
CcbHLH137	CcbHLH150	AT1G09530.2	AT2G20180.2	0.648
CcbHLH139	CcbHLH146	AT1G68920.1	AT1G68810.1	0.526
CcbHLH139	CcbHLH149	AT1G68920.1	AT3G06590.2	0.623
CcbHLH140	CcbHLH148	AT1G69010.1	AT1G29950.2	0.487
CcbHLH140	CcbHLH146	AT1G69010.1	AT1G68810.1	0.617
CcbHLH146	CcbHLH148	AT1G68810.1	AT1G29950.2	0.627

Table S7. IIIf subfamily *CcbHLHs* protein interaction analysis

Cinnamomun camphcra protein	*Arabidopsis thaliana* protein	identity	bitscore	Preferred Name
CcbHLH001	AT5G41315.1	43.2	462.2	GL3
CcbHLH001	AT1G63650.3	42.9	449.1	EGL3
CcbHLH001	AT4G00480.2	33.7	274.6	ATMYC1
CcbHLH001	AT4G09820.1	31.2	261.2	TT8
CcbHLH022	AT4G09820.1	42	387.9	TT8
CcbHLH022	AT1G63650.3	29.9	258.1	EGL3
CcbHLH022	AT5G41315.1	29.3	249.6	GL3
CcbHLH022	AT4G00480.2	22.3	142.9	ATMYC1
CcbHLH118	AT5G41315.1	39.2	395.6	GL3
CcbHLH118	AT1G63650.3	39	385.2	EGL3
CcbHLH118	AT4G00480.2	30.3	235.7	ATMYC1
CcbHLH118	AT4G09820.1	28.7	224.6	TT8
CcbHLH134	AT4G09820.1	40.3	401.7	TT8
CcbHLH134	AT5G41315.1	29.1	268.9	GL3
CcbHLH134	AT1G63650.3	30.2	261.5	EGL3
CcbHLH134	AT4G00480.2	22.4	141.7	ATMYC1

Table S8. The primers used in the qRT-PCR.

Name	Sequence
q-CcbHLH001-F	CAATCCCGTGAAGGTTGTGC
q-CcbHLH001-R	TGAAAGGGTCTTCGTGTAGTGAGG
q-CcbHLH015-F	CAGCAATAGAAACTTTGGTGGGC
q-CcbHLH015-R	TCTGCCTCGAATCCATCGGTAT
q-CcbHLH017-F	GCTGGTCGAGCTGGGTCTATTC
q-CcbHLH017-R	GCTTTATCCGTTTTGGTGGTGC
q-CcbHLH022-F	ATGGAGGTAAGCACTGAGGAGGC
q-CcbHLH022-R	CAGGTGGGAAGGAGAAGGAGACA
q-CcbHLH101-F	CTGCTCAGGTCCACAATCTTTCAG
q-CcbHLH101-R	TCATGCCACTCCCCATCCAC
q-CcbHLH118-F	GAGTTACGGTGTCTGTGGAGGG
q-CcbHLH118-R	ATGTCAACGTCAAGGTCAGGAAT
q-CcbHLH134-F	TGGATGGGGTCCTTGAACTCG
q-CcbHLH134-R	TGCAGGCTTGGGGAAGTGGT
ACT-F	GTACAGTGTCTGGATTGGAGGCTC
ACT-R	CTGCCAGTCTTCACATGATCAGAA
